# Insights into human melanocyte development and characteristics through pluripotent stem cells combined with single-cell sequencing

**DOI:** 10.1016/j.isci.2025.112373

**Published:** 2025-04-08

**Authors:** Jie Yang, Zihan Wang, Hang Zhou, Yuyun Xiong, Yumei Li, Yun-wen Zheng, Liping Liu

**Affiliations:** 1 Department of Dermatology, and Institute of Regenerative Medicine, Affiliated Hospital of Jiangsu University, Jiangsu University, Zhenjiang, Jiangsu 212001, China; 2 Changzhou Jintan First People’s Hospital, Affiliated Jintan Hospital of Jiangsu University, Changzhou, Jiangsu 213251, China; 3 Haihe Laboratory of Cell Ecosystem, Institute of Hematology, Chinese Academy of Medical Sciences, Tianjin 300020, China; 4 Guangdong Provincial Key Laboratory of Large Animal Models for Biomedicine, South China Institute of Large Animal Models for Biomedicine, and School of Pharmacy and Food Engineering, Wuyi University, Jiangmen, Guangdong 529020, China; 5 Department of Medical and Life Sciences, Faculty of Pharmaceutical Sciences, Tokyo University of Science, Tokyo 125-8585, Japan; 6 Department of Dermatology, WuHu Hospital, East China Normal University (The Second People’s Hospital, WuHu), Wuhu, Anhui 241000, China

**Keywords:** Cell biology, Stem cells research, Transcriptomics

## Abstract

Human pigment-related diseases are closely linked to melanocytes, yet our understanding of their development has largely relied on animal models. The utilization of pluripotent stem cells (PSCs) has shown immense potential in advancing our knowledge of human developmental biology. This study utilized human PSCs and single-cell sequencing to investigate the cellular heterogeneity and dynamic changes in biological characteristics, differentiation trajectory, and signaling interactions during melanocyte development. By integrating datasets from normal human melanocytes, we verified that PSC-derived melanocytes closely resemble normal human melanocytes, especially in early stages. Exploration of cell-cell communication revealed intricate signaling pathways, including Bone Morphogenetic Protein (BMP), Wingless/Integrated (WNT), and transforming growth factor β (TGF-β), in subpopulations of induced melanocytes. Additionally, PDGFRB may serve as a potential surface marker for stemness maintenance in melanocytes. Collectively, these findings demonstrate that PSCs can effectively stimulate human melanocyte development, thereby providing a valuable tool for further investigations into melanocyte-related diseases.

## Highlights

• PSCs effectively stimulate human melanocyte development, especially in early stages
• Signaling pathways, such as BMP, WNT, and TGF-β, exist among induced melanocytes
• PDGFRB may act as a potential surface marker for melanocyte stemness maintenance

## Graphical abstract

**Figure:**
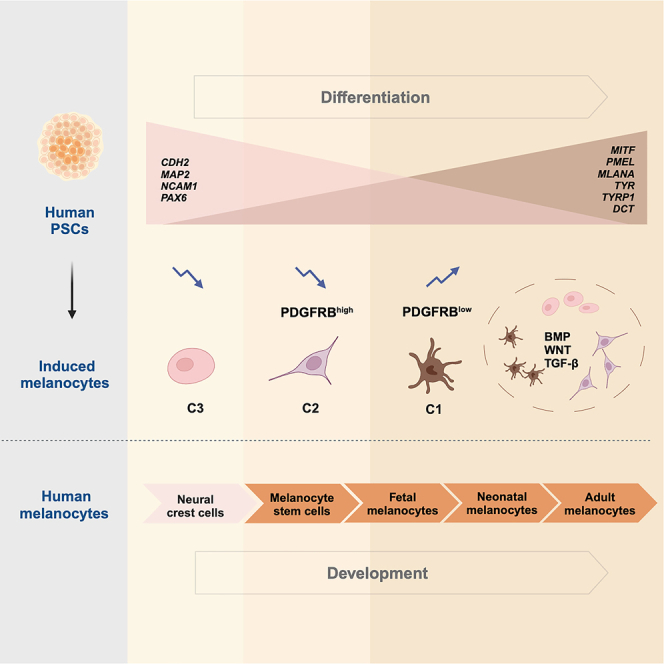

## Introduction

Melanocytes, specialized cells in the human body, are responsible for producing melanin, which protects the skin and hair against ultraviolet radiation damage. During embryonic development, these cells originate from neural crest cells (NCCs), a transient, multipotent cell population.^1^^,^^2^ NCCs undergo epithelial-to-mesenchymal transition (EMT) and migrate along distinct pathways to populate the epidermis and hair follicles.^3^^,^^4^ Melanocyte stem cells are undifferentiated melanocyte precursors with lineage-restricted differentiation potential and self-renewal capacity. These cells reside in specific niches, such as the bulge region of hair follicles, and generate melanocytes, including those in hair follicles and possibly the epidermis. Dysregulation during this migration process can lead to the retention of melanocytes in the dermis, resulting in congenital dermal melanocytosis disorders including Ota nevus, Ito nevus, Mongolian spots, etc.^5^ Furthermore, the impairment or dysfunction of melanocytes can give rise to depigmenting diseases such as albinism^6^^,^^7^ and vitiligo.^8^^,^^9^ These conditions not only impact physical appearance but also significantly affect the psychological well-being and quality of life of patients.^10^^,^^11^^,^^12^ Melanocytes are also closely associated with melanoma, the most severe form of skin malignancy.^13^^,^^14^ To explore the underlying pathogenesis of these diseases and develop more effective treatment strategies, it is crucial to comprehensively understand the dynamic process of embryonic development in melanocytes and study their unique characteristics. Given the ethical constraints on obtaining human embryonic tissue samples, animal models such as mice^15^^,^^16^ and zebrafish^7^^,^^17^ are widely utilized. However, it is important to acknowledge that there are physiological, anatomical, and metabolic differences among different species and that animal models cannot fully simulate the biological characteristics in humans.^18^^,^^19^^,^^20^ This limitation poses significant challenges in the treatment of melanocyte-related diseases.^21^^,^^22^

Pluripotent stem cells (PSCs), including embryonic stem cells and induced pluripotent stem cells (iPSCs), offer valuable *in vitro* research tools for studying the development of various human cell types due to their unlimited proliferation and potential for multi-lineage differentiation. These cells have been successfully utilized in studying lineage-specific cells such as neurons,^23^^,^^24^ muscles,^25^^,^^26^ and blood cells.^27^^,^^28^ They hold promise for providing novel treatment strategies for challenging diseases through the construction of disease models.^29^^,^^30^ In our previous studies,^31^^,^^32^ functional induced melanocytes derived from human PSCs were generated using a suspension differentiation system, and the presence of melanocyte stem cells during early differentiation stages was observed.

In this study, we combined the differentiation system with single-cell RNA sequencing (scRNA-seq) to investigate the dynamic changes in biological characteristics and signaling interactions during melanocyte differentiation. Our findings reveal that PSC-derived melanocytes follow a developmental trajectory that closely resembles human melanocytes, particularly in the early stages. These discoveries advance our understanding of melanocyte biology and offer valuable insights for improving disease modeling and therapeutic strategies for melanocyte-related disorders.

## Results

### Dynamic expression patterns of neural crest and melanocyte markers during the differentiation of PSCs into melanocytes

**Figure 1:**
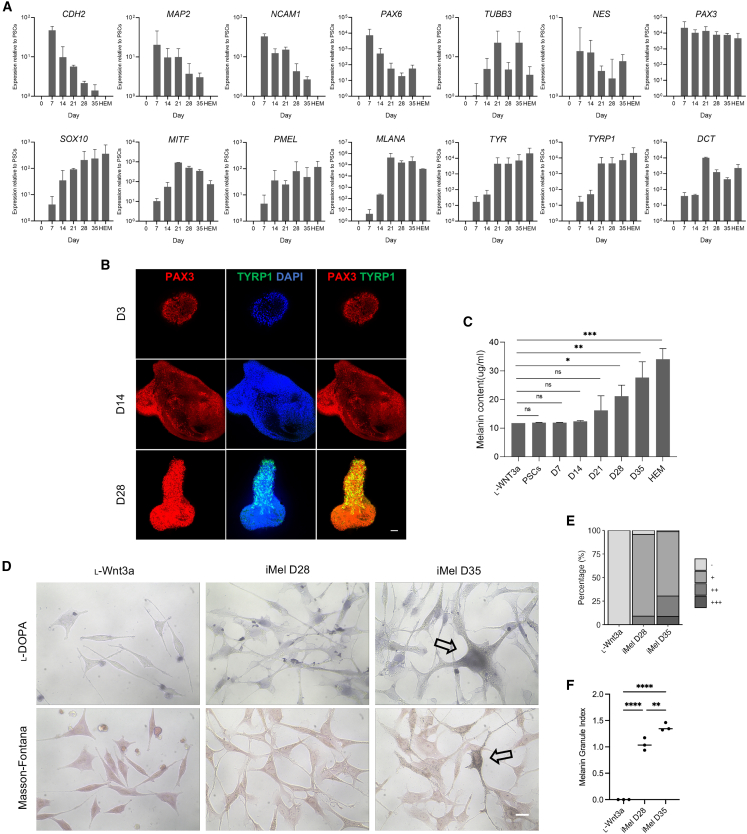
Dynamic changes in the differentiation process of PSCs into melanocytes (A) Quantitative PCR analysis of neural crest marker genes and melanocyte genes in the process of differentiation. Bars represent the relative quantity normalized to *GAPDH* (mean ± SD) relative to undifferentiated PSCs. HEM, human epidermal melanocytes. Results are calculated from three independent experiments. (B) Immunostaining of PAX3 (red) and TYRP1 (green) performed on day 3, day 14, and day 28 during differentiation. Scale bars, 50 μm. (C) Melanin content of induced melanocytes during the differentiation process. Results are calculated from three independent experiments (ns, not significant, ∗*p* < 0.05, ∗∗*p* < 0.01, ∗∗∗*p* < 0.001, Student’s t test). (D) _L_-DOPA and Masson-Fontana staining of induced melanocytes (iMel). Arrows, melanocytes with positive staining. Scale bars, 20 μm. (E) The proportion of melanin granule levels in the Masson-Fontana staining of induced melanocytes. Results are calculated from three independent experiments. (F) Quantification of the melanin granule index derived from Masson-Fontana staining. Results are calculated from three independent experiments (∗∗*p* = 0.0060, ∗∗∗∗*p* < 0.0001, one-way ANOVA).

The differentiation of PSCs into melanocytes was dynamically observed using our previously established suspension protocol.^31^^,^^32^ As melanocytes originate from NCCs, the expression of neural crest- and melanocyte-specific markers was closely monitored. On day 7 of induction, there was a significant upregulation of *CDH2*, *MAP2*, *NCAM1*, and *PAX6*, followed by a gradual decrease in their expression levels (Figure 1A). Notably, these markers are nearly undetectable in normal melanocytes. In contrast, *TUBB3*, *NES*, and *PAX3* maintained elevated levels after the initial upregulation. The expression levels of *SOX10*, *MITF*, *PMEL*, *MLANA*, *TYR*, *TYRP1*, and *DCT* steadily increased throughout the differentiation process, peaking around day 21 to day 28, and remained at a high level, comparable to those of normal human epidermal melanocytes. Immunofluorescence staining confirmed the presence of the neural crest marker PAX3 from day 3 of differentiation, while the expression of the mature marker TYRP1 was not observed during the first two weeks (Figure 1B). To assess the maturity of the induced melanocytes, melanin content was measured. Starting from day 28 of differentiation, there was a significant increase in melanin content, gradually approaching the level observed in normal melanocytes (Figure 1C). Furthermore, positive results from _L_-DOPA and Masson-Fontana staining were obtained around day 35 of differentiation, indicating the maturation of the melanocytes (Figures 1D–1F).

Overall, these findings indicate the dynamic expression patterns of neural crest and melanocyte markers during the differentiation of PSCs into induced melanocytes, along with the progressive maturation of melanocyte function.

### Unraveling the cellular heterogeneity and developmental characteristics of induced melanocytes through scRNA-seq

**Figure 2:**
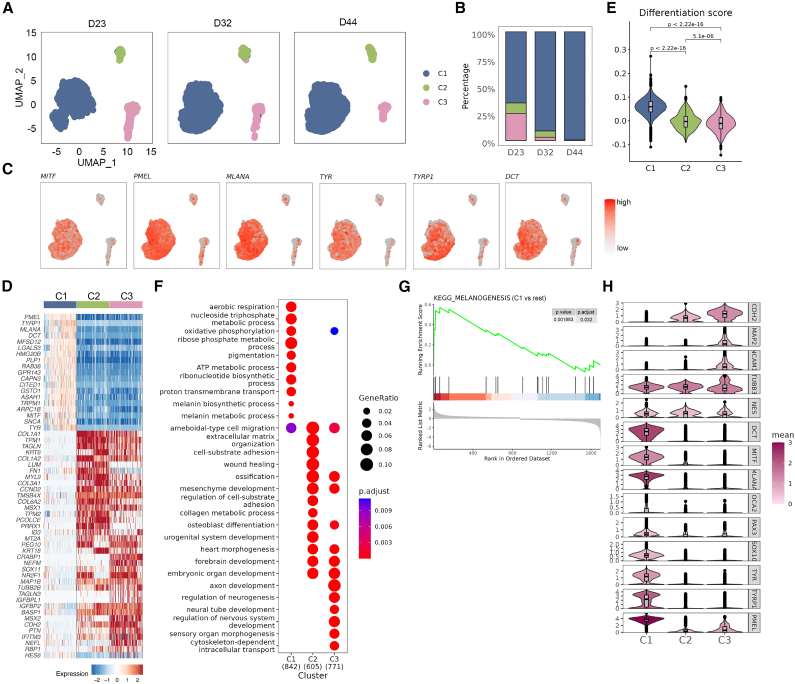
The heterogeneous characteristics of induced melanocytes at different developmental stages (A) Uniform manifold approximation and projection (UMAP) plot showing induced melanocyte (iMel) datasets split by the three time points of differentiation (D23, *n* = 3,795; D32, *n* = 6,023; and D44, *n* = 5,811). Each dot represents a cell colored by its cluster (“C”). (B) Proportion of three cell clusters at each time point. (C) Feature plots showing the expression distribution for melanocyte-specific genes. (D) Heatmap illustrating the top 20 genes enriched in each cluster. The expression level is indicated by color, blue (low) to red (high). (E) Violin plot indicating the melanocyte differentiation score^34^ in each clusters (one-sided Wilcoxon rank-sum test). (F) Dot plot showing the top 10 GO-BP terms of upregulated DEGs in each cluster. The numbers of selected genes are shown at the bottom. The size and color of each dot represent the gene ratio and significance level, respectively. (G) Gene set enrichment analysis for melanogenesis from MSigDB hallmark terms by comparing C1 with two other clusters (Benjamini-Hochberg method). (H) Violin plot showing the expression of neural crest and melanocyte genes in each cluster. See also Figure S1.

To comprehensively elucidate the molecular characteristics of human melanocytes during differentiation, scRNA-seq was performed on induced melanocytes at three distinct differentiation time points (day 23, day 32, and day 44) (Figure S1A). Following quality control (Figure S1B), 15,630 cells were included for subsequent analysis. Using integration methods from the Seurat^33^ R package, 9 clusters (c0–c8) (Figure S1C) were identified and grouped into three major populations (C1, C2, and C3) based on uniform manifold approximation and projection (UMAP) analysis (Figures 2A and S1C–S1E). The distribution of these clusters changed dynamically during differentiation, with C1 cells gradually increasing in proportion, while C2 and C3 decreased (Figures 2B and S1F). The melanocyte marker-based analysis revealed distinct characteristics across the three clusters. C1 showed high expression of melanocyte-specific genes (*DCT*, *MITF*, *MLANA*, *PMEL*, *TYR*, and *TYRP1*), indicating a differentiated melanocyte identity (Figures 2C and 2D; Table S2). Consistently, the melanocyte differentiation score,^34^ calculated using AddModuleScore, was notably higher in the C1 cluster compared to the other two clusters (Figure 2E; Table S2). Gene ontology (GO) biological process (BP) terms and the Kyoto Encyclopedia of Genes and Genomes (KEGG) enrichment analysis confirmed that upregulated differentially expressed genes (DEGs) in C1 were associated with melanin production and pigmentation, reinforcing the mature melanocyte identity of this cluster (Figures 2F and 2G; Table S2).

To explore the developmental processes of induced melanocytes, neural crest markers were analyzed. Significant enrichment of *CDH2*, *TUBB3* and *NES* was observed in both C2 and C3, while *MAP2* and *NCAM1* were specifically enriched in C3 (Figure 2H; Table S2), suggesting that C2 and C3 may represent early-stage cell populations during melanocyte development. GO-BP analysis revealed that C2 was associated with extracellular matrix organization, mesenchymal development, and embryonic organ development. While C3 shared these terms, it was particularly enriched in neural-related processes, such as axon development, the regulation of neurogenesis, neural tube development, and the regulation of nervous system development (Figure 2F). Neural-related GO-BP scoring analysis demonstrated that C3 exhibited the highest neural enrichment among the three clusters (Figure S1G; Table S2).

Taken together, these findings reveal the distinct phases of melanocyte differentiation, comprising early-stage populations (C2 and C3) and a mature melanocyte population (C1).

### Deciphering the developmental trajectory of induced melanocytes

**Figure 3:**
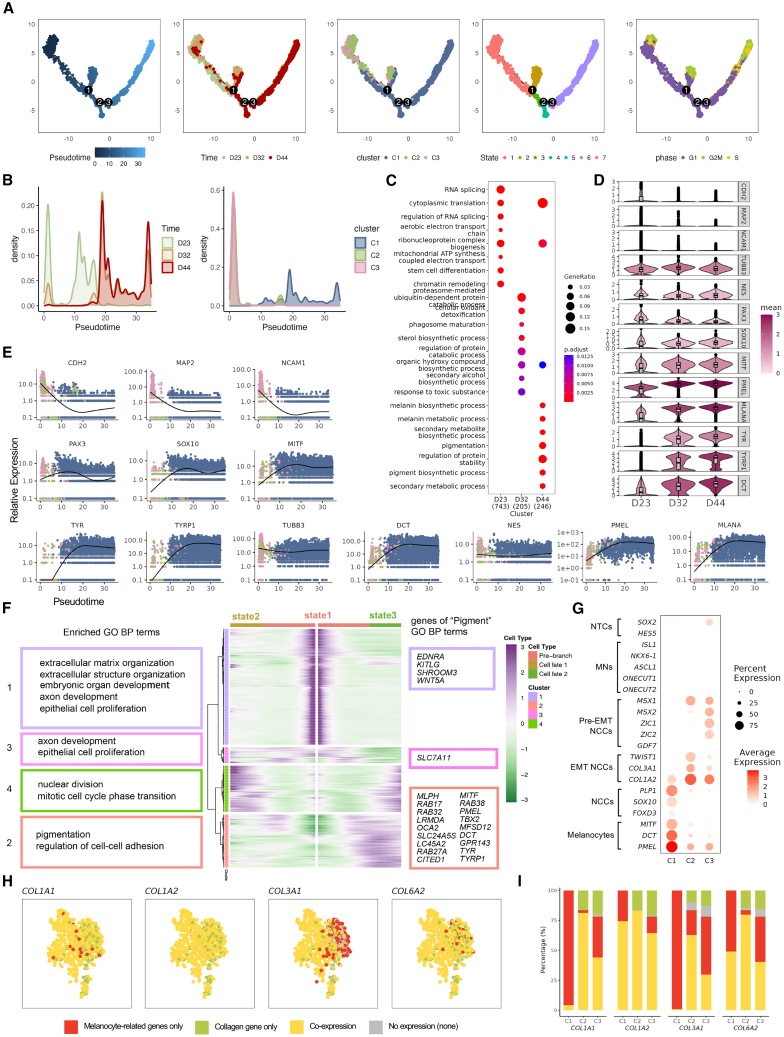
The dynamic differentiation trajectory of induced melanocytes (A) Monocle pseudo-time trajectories showing melanocyte differentiation for the pseudo-time order, time point, cell cluster, cell state, and cell cycle. Cells are colored by progression through pseudo-time and cell identities. (B) Density plots showing distribution of time points (left) and cell clusters (right) within the progression through pseudo-time. (C) Dot plot of top 8 GO-BP terms for upregulated DEGs at three time points of differentiation. (D) Violin plot showing the expression of neural crest and melanocyte genes at three time points of differentiation. (E) Genes from (D) projected onto Monocle pseudo-time trajectories. (F) Pseudo-temporal dynamics analysis of the 1,790 branch-dependent genes identified by branch expression analysis modeling showing four distinct patterns. Enriched GO-BP terms are shown in the left frames. Genes within GO-BP terms containing “pigment” are shown in the right frames. (G) Dot plot showing the expression of marker genes from neural cell types of early embryos in the three cell clusters. NTCs, neural tube cells; MNs, motor neurons; Pre-EMT NCCs, pre-epithelial-to-mesenchymal transition neural crest cells; EMT NCCs, epithelial-to-mesenchymal transition neural crest cells; NCCs, neural crest cells. (H) Enlarged UMAP plots of the C2 cluster, labeled by *COL1A1*, *COL1A2*, *COL3A1*, and *COL6A2*. Cells are colored as follows: red indicates expression of at least one melanocyte-related gene (*DCT*, *MITF*, *MLANA*, *OCA2*, *PAX3*, *SOX10*, *TYR*, *TYRP1*, and *PMEL*), yellow indicates co-expression of both melanocyte-related and collagen genes, green indicates expression of collagen genes only, and gray indicates no expression of these genes. (I) Bar plots summarizing the proportions of cells in C1, C2, and C3 based on the states defined in (H): gray (none), green (collagen only), red (melanocyte only), and yellow (co-expression of melanocyte-related and collagen genes). See also Figure S2.

To gain deeper insights into the developmental trajectory of induced melanocytes, pseudo-time analysis was performed using Monocle2.^35^^,^^36^^,^^37^ This analysis revealed the differentiation process of melanocytes in relation to pseudo-time order, time point, cell cluster, cell state, and cell cycle (Figures 3A and S2A). The results revealed that the day 23 sample (D23) predominantly occupied the early stages of the pseudo-time trajectory, whereas the day 44 sample (D44) corresponded to the later stages (Figure 3B). To characterize the molecular changes during differentiation, DEGs across the three differentiation time points (D23, D32, and D44) were analyzed. GO-BP enrichment analysis highlighted stage-specific processes, with genes associated with stem cell differentiation showing significant upregulation on D23, while genes involved in melanin biosynthesis and pigmentation were predominantly enriched on D44 (Figure 3C; Table S3). Additionally, neural crest markers peaked at earlier stages, while melanocyte markers dominated later stages (Figure 3D; Table S3), aligning with pseudo-time trajectory (Figure 3E) and qPCR results (Figure 1A). These results demonstrate that the pseudo-time trajectory accurately reflects the progression of induced melanocyte differentiation, aligning well with the temporal dynamics observed across actual differentiation time points.

Since branch 1 (Figure 3A) signifies a critical transition from state 1 (pre-branch) to states 2 and 3, a time-series heatmap was generated, revealing four gene modules (Figure 3F; Table S3). Module 1 in state 1 was enriched for “extracellular matrix organization” and “axon development,” while module 2 in state 3 was associated with pigmentation. Key genes such as *EDNRA*, *KITLG*, and *WNT5A* were downregulated during differentiation, whereas *MITF* and *TYR* exhibited high expression levels in state 3. The data in Figures 3A and 3F indicate that state 2 corresponds to early differentiation, while state 3 represents a more mature melanocyte state. Although cell cycle effects may influence the differences between states 2 and 3, branch 1 delineates a progression from progenitor-like states to mature melanocytes.

In addition, pseudo-time analysis revealed that C2 and C3 predominantly occupied the early stages of the trajectory, transitioning into C1 as differentiation progressed (Figures 3A and 3B), aligning with the findings in Figure 2. To clarify the sequential relationship between C2 and C3 in the pseudo-time analysis, marker genes from neural cell types of early embryos^38^ were introduced. The results revealed that C3 corresponded to pre-EMT NCCs characterized by *MSX1*, *MSX2*, *ZIC1*, and *ZIC2*, while C2 corresponded to EMT NCCs marked by *TWIST1*, *COL3A1*, and *COL1A2*. C1 represented NCCs transitioning into melanocytes, indicated by *PAX3*, *SOX10*, and *FOXD3* (Figure 3G; Table S4). Furthermore, C3 can be separated into two clusters (Figure S2B), representing pre-EMT NCCs and neural tube cells (NTCs). Compared to pre-EMT NCCs, upregulated DEGs (392 genes) in the NTCs cluster were significantly enriched in GO-BP related to axon development, forebrain development, regulation of neurogenesis, and neural precursor cell proliferation (Figure S2C; Table S4). Notably, all these GO-BP-related genes exhibited high expression levels in the NTCs population of C3 compared to other clusters (Figures S2D–S2G; Table S4). Given that NCCs are derived from NTCs and pre-migratory NCCs,^38^ and that the proportion of both NTCs and pre-EMT NCCs clusters decreased during the differentiation process (Figure S2H), C3 likely represents an earlier stage of cells in the melanocyte differentiation process relative to other clusters.

Because C2 and C3 exhibited high expression of collagen genes, including *TWIST1*, *COL3A1*, and *COL1A2*, co-expression analysis was conducted to confirm that they were not fibroblasts. More than 75% of cells in the C2 and C3 populations express at least one melanocytic gene, with the majority co-expressing both melanocyte and collagen genes (Figures 3H, 3I, S2I, and S2J; Table S5). Additionally, marker genes from all cell types in human skin^39^ were included, further distinguishing C2 and C3 from other cell types (Figure S2K; Table S5). Given that collagen genes are involved in EMT, which plays a pivotal role in NCCs differentiation,^2^^,^^3^^,^^38^^,^^40^ the co-expression observed in C3 and C2 suggested they may be undergoing pre-EMT and EMT, respectively.

In summary, these findings indicate that C2 and C3 represent early stages of differentiation, with C3 positioned above C2 in the hierarchy. The co-expression of melanocyte and collagen genes suggests a role for EMT in NCC migration and melanocyte differentiation.

### Comparative insights into the developmental similarity of induced and human melanocytes

**Figure 4:**
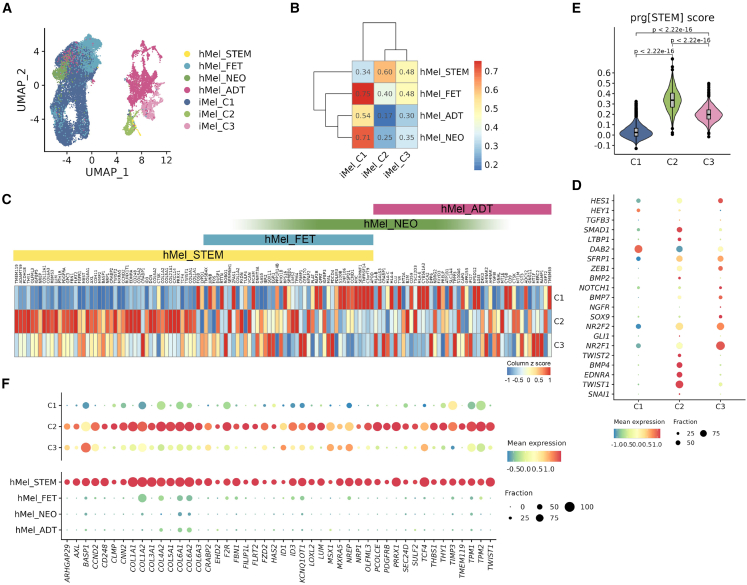
Developmental comparison of induced melanocytes derived from PSCs with human melanocytes (A) UMAP plot showing the integration of the human melanocyte^41^ (hMel) and induced melanocytes (iMel) datasets. STEM, melanocyte stem cells; FET, fetal melanocytes; *NEO*, neonatal melanocytes; ADT, adult melanocytes. (B) Spearman correlation analysis of cell types between iMel and hMel using the top 100 DEGs of each cluster in the hMel. Prg, program; STEM, melanocyte stem cells; FET, fetal melanocytes; *NEO*, neonatal melanocytes; ADT, adult melanocytes. (C) Heatmap showing the relative expression (column *Z* score) of specific genes identified from four hMel developmental stages in three iMel clusters. (D) Dot plot showing the relative expression of melanocyte stem cell markers^42^^,^^43^^,^^44^ in three iMel clusters. (E) Violin plots illustrating the stemness score calculated from the specific genes enriched in the melanocyte stem cells program in hMel, as identified in (C) (one-sided Wilcoxon rank-sum test). (F) Dot plot indicating the conserved genes in the three clusters of iMel and four developmental stages of hMel. See also Figures S3 and S4.

Induced melanocytes derived from PSCs offer significant potential for studying human melanocyte development and associated diseases. Assessing their molecular and developmental similarity to native human melanocytes is essential to validating their value as an *in vitro* model. Therefore, scRNA-seq dataset from human skin melanocytes^41^ was utilized, where melanocytes were categorized into four developmental stages: melanocyte stem cells, fetal, neonatal, and adult. Integration of scRNA-seq datasets from human melanocytes and induced melanocytes using Seurat^33^ revealed that C1 in the induced melanocytes encompassed the fetal, neonatal, and adult stages of human melanocytes, while C2 aligned more closely with the melanocyte stem cells (Figures 4A and S3A). Notably, C3 appeared distinct and did not overlap with any specific stages of human melanocytes.

Correlation analysis based on the top 100 DEGs from the four developmental stages of human melanocytes showed that C1 displayed higher similarity to fetal and neonatal melanocytes (r = 0.75 and 0.71, respectively), whereas C2 exhibited greater similarity to melanocyte stem cells (r = 0.60). In contrast, C3 showed low correlation with any stage of human melanocytes (Figures 4B and S3B; Table S6). Furthermore, C1 exhibited concurrent expression of DEGs associated with fetal, neonatal, and adult stages, while DEGs upregulated in melanocyte stem cells were significantly enriched in the C2 cluster (Figure 4C; Table S6).

Detailed analysis of C1 revealed internal heterogeneity, evident from its six distinct subclusters (Figure S3C). Genes associated with melanocyte stem cells and fetal melanocytes were enriched in C1b, while genes associated with the neonatal and adult stages were relatively enriched in C1d (Figures S3D and S3E; Table S6). GO-BP analysis further suggested potential differences in melanocyte differentiation states among these subclusters (Figure S3F; Table S6). To validate the stem cell characteristics of C2, highly variable genes from established melanocyte stem cell signatures reported in previous studies^42^^,^^43^^,^^44^ were analyzed, revealing significant enrichment in C2 (Figure 4D; Table S6). Furthermore, the stemness score, as described by Belote et al.,^41^ was also highest in C2 (Figure 4E; Table S6). Additionally, a group of conserved genes that were enriched in both human melanocyte stem cells and C2 were identified (Figure 4F; Table S6). GO-BP analysis indicated that these genes were associated with “extracellular matrix organization” and “response to TGF-β signaling pathways,” aligning with the characteristics of melanocyte stem cells (Figure S3G; Table S6).

Mouse and human melanocytes share significant similarities, and much of our understanding of melanocyte characteristics is based on studies of mouse melanocytes. To assess whether induced melanocytes more closely resemble human melanocytes than mouse melanocytes, scRNA-seq datasets of mouse melanocytes^16^^,^^45^ were incorporated. In these studies, mouse melanocytes were classified into telogen melanocyte stem cells, early-anagen melanocyte stem cells, and differentiated melanocytes. Stemness score^41^ and differentiation score,^34^ calculated using human melanocyte genes, aligned mouse melanocyte states with those described in the original studies^16^^,^^45^ (Figure S4A; Table S7). Notably, telogen melanocyte stem cells exhibited the highest stemness score, while differentiated melanocytes displayed the highest differentiation score. However, these characteristics of mouse melanocytes did not adequately capture the distinctions of the four developmental stages of human melanocytes (Figure S4B; Table S7) as well as induced melanocytes did (Figure 4C; Table S6).

Pearson correlation analysis revealed greater similarity between induced melanocytes and human melanocytes (0.78), compared to mouse melanocytes and human melanocytes (0.7) (Figure S4C). Further clustering analysis divided melanocytes from the three sources into differentiated and undifferentiated groups. Hierarchical clustering analysis of melanocyte-related genes revealed that human and induced melanocytes clustered together, indicating greater similarity compared to mouse melanocytes (Figure S4D; Table S7). Venn diagrams of upregulated DEGs further confirmed this, demonstrating greater overlap between human melanocytes and induced melanocytes than between human and mouse melanocytes (30.1% vs. 24.6% in undifferentiated melanocytes and 65.8% vs. 36.8% in differentiated melanocytes) (Figure S4E; Table S7).

These findings indicate that induced melanocytes derived from human PSCs follow a developmental trajectory that closely resembles human melanocytes. Specifically, C1 corresponds to differentiated melanocytes, particularly at fetal and neonatal stages, while C2 aligns with melanocyte stem cells.

### Revealing the cell-cell interactions and signaling in melanocyte differentiation

**Figure 5:**
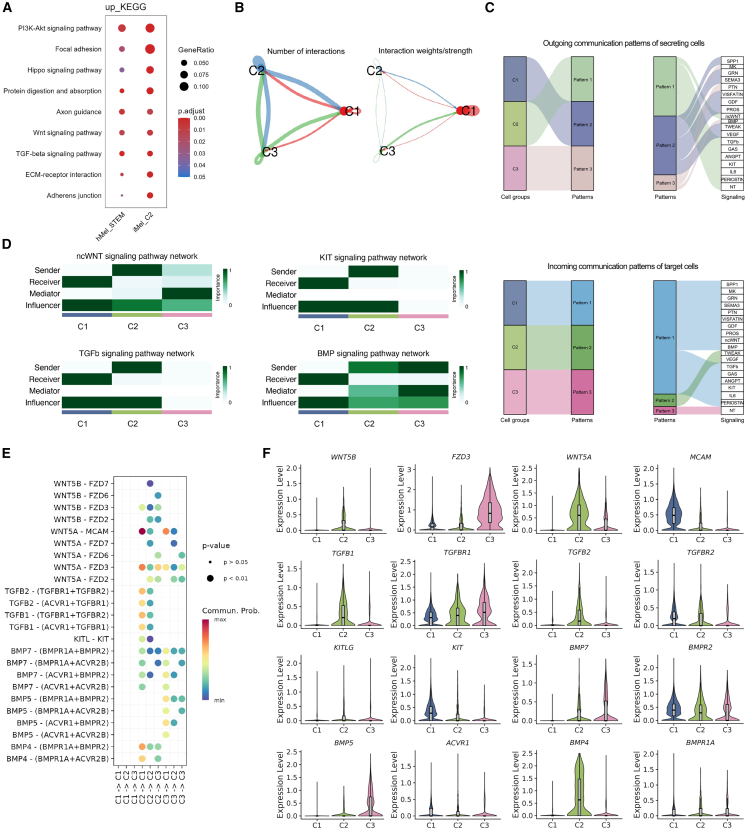
Cell-cell signaling in induced melanocytes (A) Dot plot displaying specific common KEGG pathways indicated by differentially expressed genes, respectively, in the C2 cluster from the induced melanocytes (iMel) dataset and stem cells from the human melanocyte (hMel) dataset. (B) Circle plots showing the number of interactions (left) and the total interaction weights/strength (right) between any two cell clusters of iMel. (C) The inferred outgoing (above) and incoming (below) communication patterns of target cells. The thickness of the flow indicates the contribution of the cell group or signaling pathway to each latent pattern. (D) Heat maps showing the relative importance of each cluster based on the computed four network centrality measures of ncWNT, KIT, TGF-β, and BMP signaling. (E) Dot plot of the ncWNT, KIT, TGF-β, and BMP signaling ligand-receptor pairs that contribute to the signaling from one cluster to another. The dot color and size represent the calculated communication probability and *p* values, respectively; the *p* values are computed using a one-sided permutation test. (F) Violin plots showing the average expression of selected ligands and receptors from (E) in the three clusters of induced melanocytes.

Cell-cell interactions and signaling pathways play an essential role in maintaining the quiescence of stem cells and regulating the process of differentiation. To investigate the signaling pathways and fate regulation involved in melanocyte differentiation, the upregulated DEGs in both human normal melanocyte stem cells and C2 of induced melanocytes were analyzed. These cells exhibited similar expression patterns, with the upregulation of signaling pathways such as Wingless/Integrated (WNT) and TGF-β, which are well-established in maintaining melanocyte stemness and regulating differentiation^15^^,^^46^^,^^47^^,^^48^ (Figure 5A; Table S8). To further explore the specific cell-cell interactions, analysis using CellChat^49^ revealed that C2 exhibited the highest number of interactions, while C1 demonstrated the strongest interaction strength (Figure 5B). Incoming and outgoing signaling communication patterns analysis showed that C2 and C3 were predominant cells generating outgoing signals, with C2 outputting non-canonical WNT (ncWNT), TGF-β, and KIT signals, and C3 focusing on Bone Morphogenetic Protein (BMP) signals. In contrast, C1 was the predominant recipient cells of these signals (Figures 5C and 5D). Ligand-receptor interactions analysis demonstrated that C2 mainly interacted with C1 through signature genes such as *WNT5A*, *TGFB1*, *TGFB2*, *BMP4*, and *KITLG*, while C3 interacted with C1 primarily via *BMP5* and *BMP7* (Figures 5E and 5F). Notably, the TGF-β and KIT ligand-KIT (KITL-KIT) pathways were specifically observed in interaction between C2 and C1, as well as within C2 itself. These signaling pathways, recognized for their role in maintaining the quiescence of melanocyte stem cells,^15^^,^^46^^,^^48^ suggested that C2 may regulate its own stemness homeostasis through these signals while also influencing the differentiation process of C1.

In summary, the analysis of cell-cell communication patterns unveils the intricate interactions and regulatory mechanisms governing melanocyte differentiation.

### Screening of potential surface markers for stemness maintenance in melanocytes

**Figure 6:**
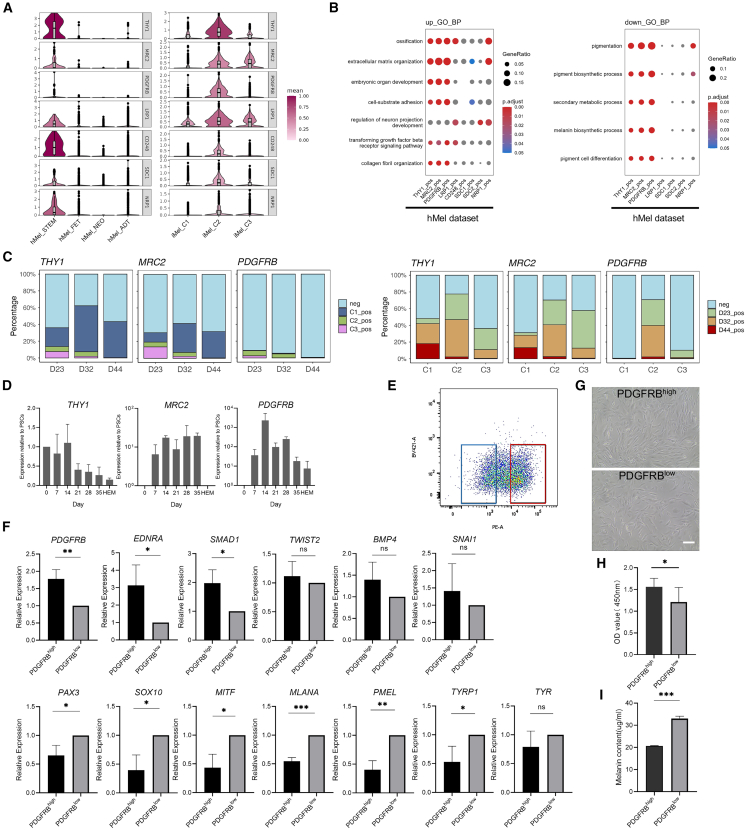
Screening of surface markers for stemness maintenance in melanocytes (A) Violin plots showing surface marker genes in human melanocyte (hMel) and induced melanocytes (iMel) datasets. STEM, melanocyte stem cells; FET, fetal melanocytes; NEO, neonatal melanocytes; ADT, adult melanocytes. (B) Dot plots showing the selected biological process terms in gene ontology for the upregulated and downregulated DEGs of the surface marker positive clusters in the hMel dataset. pos, positive. (C) Bar plots showing the proportion of positive clusters of selected surface markers in three clusters at three time points and at three time points in three cell clusters, respectively. neg, negative; pos, positive. (D) Quantitative PCR analysis showing the dynamic expression of screened surface marker genes in the process of induced melanocyte differentiation. The bars represent the relative quantity normalized to GAPDH (mean ± SD) relative to undifferentiated PSCs calculated from three independent experiments. (E) Fluorescence-activated cell sorting of induced melanocytes (blue frame: PDGFRB^low^; red frame: PDGFRB^high^). (F) Quantitative PCR analysis showing a gene expression comparison of melanocyte markers and melanocyte stem cell markers between the PDGFRB^high^ and PDGFRB^low^ populations. The bars represent the relative quantity normalized to that of GAPDH (mean ± SD) relative to the PDGFRB^low^ group calculated from three independent experiments (ns, not significant, ∗*p* < 0.05, ∗∗*p* < 0.01, ∗∗∗*p* < 0.001, Student’s t tests). (G) Culture of induced melanocytes for 48 h after sorting with the same seeding density. Scale bar, 100 μm. (H) Cell proliferation assay using Cell Counting Kit-8 showing the OD value after 24 h of culture after sorting. Results are calculated from three independent experiments (mean ± SD) (∗*p* < 0.05, Student’s t tests). (I) Comparison of melanin content between the PDGFRB^high^ and PDGFRB^low^ cell populations after sorting. Results are calculated from three independent experiments (mean ± SD) (∗∗∗*p* < 0.001, Student’s t tests). See also Figure S5.

The investigation of surface markers could facilitate the screening and enrichment of melanocyte subpopulations that exhibit stemness characteristics. Comparative analysis of DEGs in human melanocyte stem cells and C2 of induced melanocytes, 210 shared genes were identified (Figure S5A; Table S9), including eight surface marker genes: *THY1*, *MRC2*, *PDGFRB*, *LRP1*, *CD248*, *SDC1*, *SDC2*, and *NRP1*. The expression levels and proportions of positive cells for these markers were significantly elevated in both human melanocyte stem cells and C2 compared to other subclusters (Figures 6A and S5B; Table S9). Further analysis of human melanocyte stem cells revealed that upregulated DEGs in *THY1*-, *MRC2*-, and *PDGFRB*-positive populations were associated with GO-BP terms such as extracellular matrix organization, embryonic organ development, cell-substrate adhesion, and the TGF-β receptor signaling pathway (Figures 6B and S5C; Table S9). Conversely, downregulated DEGs in these populations were linked with pigmentation-related GO-BP terms and KEGG pathways. These findings suggest that THY1, MRC2, and PDGFRB may serve as promising surface markers for identifying melanocyte stemness.

Considering the gradual decline in surface marker expression during stem cell differentiation, the expression levels of *THY1*, *MRC2*, and *PDGFRB* were examined in induced melanocytes. Among these markers, *PDGFRB*-positive populations demonstrated a progressive decline throughout the differentiation process, predominantly expressed in C2 (Figure 6C; Table S9). This observation was validated by quantitative PCR analysis, while *THY1* and *MRC2* did not exhibit similar expression treads (Figure 6D). Subsequently, flow cytometry was employed to isolate the PDGFRB^high^ cell population, which exhibited elevated expression of melanocyte stem cell-related genes and reduced expression of mature melanocyte genes, compared to PDGFRB^low^ population (Figures 6E and 6F). Furthermore, PDGFRB^high^ populations displayed enhanced proliferative capacity (Figures 6G, 6H, and S5D) and lower melanin content (Figures 6I and S5E).

Collectively, these findings suggest that PDGFRB might be a potential marker for screening melanocyte subpopulations that maintain stemness; however, further validation is needed.

## Discussion

Understanding the origin and development of human melanocytes has primarily relied on studies on avian and rodent animal models.^50^^,^^51^^,^^52^^,^^53^ Although the fundamental biology across model organisms is largely similar, there are notable differences. Consequently, there is still a dearth of information regarding human melanocyte development. The application of PSC research in the realm of human developmental biology offers promising prospects to address this knowledge gap. Notably, Cohen et al.^54^ successfully implanted human NCCs derived from PSCs into mouse embryos, establishing a human-mouse chimeric model that validated the contribution of human NCCs to the melanocyte lineage. Recently, the emergence of single-cell sequencing technology has revolutionized biological research, enabling unprecedented levels of resolution and scalability. This advancement has facilitated a comprehensive understanding of transcriptional distinctions within cell populations. Several studies employing single-cell sequencing have examined human skin tissues, shedding light on the detailed characteristics of melanocytes.^55^^,^^56^^,^^57^ However, only a few have focused on the early stage of melanocyte development. In our study, we utilized single-cell sequencing to demonstrate that C1 of induced melanocytes effectively mimics the fetal, neonatal, and adult stages of human melanocytes derived from skin samples. Additionally, we found that C2 closely resembles melanocyte stem cells. Furthermore, C3 displayed neural crest features characteristic of the NTCs and Pre-EMT stage, potentially representing an earlier population preceding the migration of melanocytes into the skin. Given the ethical concerns, these results are almost unattainable using human tissue samples. Therefore, these findings highlight the immense value of PSCs as an indispensable tool for studying the development of human melanocytes, with a specific focus on simulating early-stage melanocyte development.

The insufficient maturity of differentiated cells derived from PSCs has raised concerns regarding their safety and efficacy in clinical applications.^58^^,^^59^ For instance, cardiac myocytes,^60^ cortical neurons,^23^ and hepatocytes^61^ derived from PSCs often exhibit maturity levels comparable to embryonic stages. Similarly, hair follicles in skin organoids derived from PSCs only reach the developmental levels resembling the mid-pregnancy stage.^62^ However, in this study, we made a noteworthy observation that induced melanocytes derived from PSCs exhibit a wide spectrum of maturity levels. Specifically, certain subpopulations of induced melanocytes reach maturity levels comparable to adult melanocytes. This finding holds great promise for future transplantation therapies targeting pigmentary disorders as it provides strong support for the development of mature melanocytes derived from PSCs. Moreover, the incorporation of the potential surface marker PDGFRB facilitates the sorting of melanocytes in a low differentiation state from the mature cell population. This is beneficial for specific research objectives, such as studying the characteristics of melanocyte stem cells. However, employing additional effective surface markers alongside PDGFRB may provide a more precise method for screening melanocyte stem cells in future studies.

The microenvironment surrounding stem cells consists of a complex network of signals-including autocrine, paracrine, and extracellular matrix signals-that collectively influence the behavior and destiny of stem cells.^63^^,^^64^ In the context of melanocytes, TGF-β has been shown to promote the immaturity and quiescence of melanocytes by downregulating MITF, a major transcription factor involved in melanocyte differentiation, along with key genes associated with terminal melanocyte differentiation. Conversely, KITL can counteract the effects of TGF-β, preventing melanocyte apoptosis.^46^^,^^48^ In our study, we found that the C2 population generates both TGF-β and KITL signals, which may act through autocrine signaling, influencing their own behavior, and through paracrine signaling, influencing the C1 population. In addition, we observed that BMP4 is exclusively produced by the population C2, while BMP5 and BMP7 are produced by both C2 and C3. These findings align with the well-established regulatory roles of the BMP family in the development of the NCCs and melanocyte differentiation.^15^^,^^65^^,^^66^^,^^67^^,^^68^^,^^69^ Moreover, the classical WNT pathway has been implicated in the induction and determination of neural crest fate^52^^,^^70^^,^^71^ as well as their subsequent differentiation into melanocytes.^52^^,^^72^ Interestingly, we found that the C2 cluster can exert its effects on other cell subpopulations through noncanonical WNT signaling, primarily mediated by WNT5A. Notably, WNT5A is known to inhibit melanin synthesis by antagonizing the classical WNT signaling pathway.^73^^,^^74^ Therefore, these results not only suggest the existence of diverse signaling pathways that interact with and regulate different subpopulations of induced melanocytes but also validate the differentiation model of PSCs as a reflection of the intricate regulatory mechanisms involved in the fate transition of human melanocytes.

In summary, by combining *in vitro* differentiation models of PSCs with single-cell sequencing technology, this study has highlighted the heterogeneity and dynamic changes within subpopulations of human melanocytes during differentiation. PSCs have proven to be a valuable tool for investigating human melanocytes, particularly in early-stage melanocyte development. These research findings not only contribute to a deeper understanding of human melanocytes in both physiological and pathological states but also provide valuable tools and insights for exploring the underlying mechanisms of melanocyte-related diseases, such as vitiligo and melanoma.

### Limitations of the study

Although the three clusters of induced melanocytes can simulate the developmental trajectory of melanocytes, these results are derived from comparisons with other single-cell sequencing data related to NCCs and melanocyte stem cells. Precisely defining NCCs and melanocyte stem cells relies not only on their molecular characteristics but also on their spatial localization during development. Therefore, the reliance on *in vitro* culture systems may limit the generalizability of some findings. In addition, incorporating additional samples, especially earlier time points, in future studies will provide more detailed information in melanocyte development. The regulatory interactions among the three clusters were derived from *in vitro* cell communication analyses, their specific roles and relevance *in vivo* remain to be fully elucidated. This is particularly important given the spatial and temporal constraints of melanocyte stem cell development in natural contexts.

## Resource availability

### Lead contact

Requests for further information and resources should be directed to and will be fulfilled by the lead contact, Liping Liu (liuliping@ujs.edu.cn).

### Materials availability

This study did not generate new unique reagents.

### Data and code availability

• The scRNA-seq data reported in this paper have been deposited at GEO: GSE268454 and are publicly available as of the date of publication. The healthy human skin scRNA-seq dataset was obtained from the publicly accessible repository: GEO: GSE151091.^41^ The mouse melanocyte scRNA-seq datasets were obtained from the following publicly available sources: GEO: GSE113502 and GEO: GSE203051.^16^^,^^45^
• The code for the scRNA-seq analysis conducted during this study is available on GitHub at the following link: https://github.com/IOKYA/iMel_iPS_scRNA. Accession numbers are listed in the key resources table.
• Any additional information required to reanalyze the data reported in this paper is available from the lead contact upon request.

## Acknowledgments

This research was funded by the 10.13039/501100001809National Natural Science Foundation of China (82103766 and 82270697), Jiangsu Provincial Medical Key Discipline Cultivation Unit, China (JSDW202229), Haihe Laboratory of Cell Ecosystem Innovation Fund (HH24KYZX0008), China, the 10.13039/501100012245Science and Technology Planning Project of Guangdong Province of China (2021B1212040016), 10.13039/501100021171Guangdong Basic and Applied Basic Research Foundation, China (2023A1515012574).

## Author contributions

Conceptualization, L.L. and Y.Z.; methodology, L.L., J.Y., Z.W., and H.Z.; resources, L.L., Y.L., and Y.Z.; cell culture, Z.W., H.Z., and L.L.; bioinformatic analyses, J.Y.; formal analysis, J.Y., Z.W., and H.Z.; writing – original draft, J.Y., Z.W., and L.L.; writing – review and editing, all authors; funding acquisition, L.L., Y.L., and Y.Z.; supervision, L.L., Y.L., and Y.Z.

## Declaration of interests

The authors declare no competing interests.

## STAR★Methods

### Key resources table

**Table:** 

REAGENT or RESOURCE	SOURCE	IDENTIFIER
**Antibodies**		

Rabbit Polyclonal anti-PAX3	Sigma-Aldrich	Cat#HPA063659; RRID: AB_2685080
Mouse Monoclonal anti-TYRP1 (clone TA99)	Millipore	Cat#MABC592; RRID: AB_3683565
PE Mouse Anti-Human CD140b	BD Pharmingen	Cat#558821; RRID: AB_397132
Alexa Fluor 488 Goat anti-Mouse IgG1	Thermo Fisher Scientific	Cat#A21121; RRID: AB_2535764
Cy3 Goat Anti-Rabbit IgG (H+L)	Jackson ImmunoResearch Labs	Cat#111-165-144; RRID: AB_2338006

**Chemicals, peptides, and recombinant proteins**		

mTeSR	StemCell Technologies, Inc.	Cat#85850
DMEM low-glucose	GIBCO	Cat#10567014
Matrigel	Corning	Cat#354277
AggreWell EB Formation Medium	StemCell Technologies	Cat#05893
MCDB 201 Medium	Sigma-Aldrich	Cat#M6770
Non-Essential Amino Acids Solution	GIBCO	Cat#11140050
Basic fibroblast growth factor (bFGF)	Wako	Cat#068-04544
Dexamethasone	Sigma-Aldrich	Cat#D4902
Insulin-transferrin-selenium	Sigma-Aldrich	Cat#I3146
Linoleic acid-bovine serum albumin	Sigma-Aldrich	Cat#L9530
12-O-tetradecanoyl-phorbol 13-acetate (TPA)	Sigma-Aldrich	Cat#P8139
L-ascorbic acid	Sigma-Aldrich	Cat#A4403
Stem cell factor (SCF)	R & D	Cat#255-SC-050
Endothelin-3	ENZO	Cat#ALX-155-003-PC05
Cholera toxin	Sigma-Aldrich	Cat#C8052
STEM-CELLBANKER	AMSBIO	Cat#11924
Fetal bovine serum (FBS)	GIBCO	Cat#A3260802
Y27632	Wako	Cat#253-00513
ACCUTASE™	Innovative Cell Technologie	Cat#AT104-500
TrypLE Express	GIBCO	Cat#12604013
_L_-DOPA	Bomei	Cat#DD1054
Synthetic melanin	Sigma-Aldrich	Cat#M8631

**Critical commercial assays**		

RevertAid First Strand cDNA Synthesis Kit	Thermo Fisher Scientific	Cat#K1622
SYBR Premix Ex Taq Kit	Takara	Cat#RR420A
Masson-Fontana Kit	Solarbio	Cat#G2032
Ce3D™ Tissue Clearing Kit	BioLegend	Cat#427701
CCK-8 Cell Counting Kit	Vacyme	Cat#A311-02

**Deposited data**		

Single-cell RNA-seq data of induced melanocytes	This paper	GEO: GSE268454
Single-cell RNA-seq data of healthy human skin	Belote et al.^41^	GEO: GSE151091
Single-cell RNA-seq data of mouse melanocytes (telogen stage)	Sun et al.^45^	GEO: GSE113502
Single-cell RNA-seq data of mouse melanocytes (early-anagen and differentiated stages)	Sun et al.^16^	GEO: GSE203051

**Experimental models: Cell lines**		

_L-_Wnt-3a cells	ATCC	CRL-2647; RRID:CVCL_0635
WA09 (H9)	Wicell	N/A
human iPSCs	lab-owned^31^	N/A

**Oligonucleotides**		

Primers for Real-time PCR, see Table S1	This paper	N/A

**Software and algorithms**		

GraphPad Prism 8	GraphPad Software	RRID: SCR_002798
Fiji	ImageJ	http://fiji.sc; RRID: SCR_002285
Cell Ranger v6.1.2	10× Genomics	https://support.10xgenomics.com/; RRID: SCR_017344
Seurat R package v3.1.1	Stuart et al.^33^	https://satijalab.org/seurat/; RRID: SCR_016341
Harmony R package v1.1.0	Korsunsky et al.^75^	https://github.com/immunogenomics/harmony; RRID: SCR_022206
clusterProfiler R package v4.6.0	Wu et al.^76^	http://bioconductor.org/packages/release/bioc/html/clusterProfiler.html; RRID: SCR_016341
msigdbr R package v7.5.1	Liberzon et al.^77^	https://cran.r-project.org/package=msigdbr; RRID: SCR_022870
Monocle R package v2.26.0	Trapnell et al.^35^; Qiu et al.^36^^,^^37^	http://cole-trapnell-lab.github.io/monocle-release/docs/; RRID: SCR_016339
pheatmap R package v1.0.12	N/A	https://CRAN.R-project.org/package=pheatmap; RRID: SCR_016418
BiomaRt R package v2.54.0	Smedley et al.^78^	https://bioconductor.org/packages/biomaRt/; RRID: SCR_019214
CellChat R package v1.6.1	Jin et al.^49^	https://github.com/sqjin/CellChat; RRID: SCR_021946
ggplot2 R package v3.5.1	N/A	https://cran.r-project.org/web/packages/ggplot2/index.html; RRID: SCR_014601
ggpubr R package v0.5.0	N/A	https://CRAN.R-project.org/package=ggpubr; RRID: SCR_021139
R v4.2.2	R	https://cran.r-project.org/; RRID: SCR_001905
RStudio v2022.02.1	RStudio	https://posit.co/; RRID: SCR_000432
Code and analysis scripts	This paper	https://github.com/IOKYA/iMel_iPS_scRNA

**Other**		

Elplasia™ 24-well plate	Corning	Cat#4440
Ultra-low attachment plates (96-well)	Corning	Cat#3474

### Experimental model and study participant details

#### Cell lines

The iPSC line was previously established from mesenchymal stem cells obtained from the umbilical cord of a healthy male.^31^ The WA09 cell line was obtained from WiCell, prior to the signing of the Material Transfer Agreement. Detailed culture methods are described in method details. _L_-Wnt-3a cells were obtained from ATCC and was cultured in Dulbecco’s modified Eagle’s medium (Thermo Fisher Scientific) containing 10% fetal bovine serum (Thermo Fisher Scientific). No mycoplasma contamination was detected.

### Method details

#### Differentiation system of induced melanocytes

In this study, iPSCs and WA09 human ESCs were used. Melanocyte differentiation from PSCs was conducted following a previously published protocol.^31^^,^^32^ Briefly, PSCs were maintained in a feeder-free culture system with mTeSR on Matrigel. Single-cell suspensions of PSCs were prepared using ACCUTASE™. Approximately 5×10^5^ cells were seeded in each well of an Elplasia™ plate (24-well), and Y27632 was added to a final concentration of 10 μM. AggreWell EB Formation Medium was used. After 24 hours, the resulting aggregates were collected and further cultured in ultra-low attachment plates for 5-10 days. The EBs were then collected and transferred to melanocyte differentiation medium, which contained 50% conditioned medium from _L_-Wnt3a cells (ATCC), 30% low-glucose DMEM, 20% MCDB 201 medium, 0.05 μM Dexamethasone, 1×insulin-transferrin-selenium, 1×linoleic acid-bovine serum albumin, 10^−4^ M L-ascorbic acid, 50 ng/mL SCF, 100 nM Endothelin-3, 20 pM cholera toxin, 50 nM 12-O-tetradecanoyl-phorbol 13-acetate, and 4 ng/mL bFGF. The cultures were maintained in ultra-low attachment plates, with half of the medium replaced every 2-3 days. After two weeks, the differentiated EBs were collected and plated on a fibronectin-coated plate. The cells were cultured for another 7 days and then dissociated using TrypLE Express Enzyme and passaged. Differentiation medium with 0.5% FBS was used, but without TPA. Subsequently, the induced melanocytes were passaged every 3-4 days.

#### Real-time PCR analysis

Total RNA was isolated from the samples using TRIzol reagent. The isolated RNA was then reverse transcribed into cDNA using a RevertAid First Strand cDNA Synthesis Kit, following the manufacturer’s instructions. The real-time PCR amplification was performed on an Applied Biosystems QuantStudio 3 system. The SYBR Premix Ex Taq kit was used for the qPCR reactions, as per the manufacturer’s protocol. The gene expression levels were normalized to the endogenous control gene, GAPDH. The relative quantification of gene expression was calculated using the ΔΔCt method. Primers used were listed in Table S1.

#### Immunofluorescence staining

The cell spheres were first collected and washed twice with PBS to remove the culture medium. They were then fixed using a 4% paraformaldehyde solution for 20 minutes. After fixation, the spheres underwent two rounds of washing with 500 μL of washing buffer from the Ce3D™ Tissue Clearing Kit (BioLegend). Each washing step was performed on a shaking platform at room temperature for 30 minutes. Next, the fixed and washed spheres were incubated in blocking buffer on a shaking platform for 2 days. The primary antibodies were diluted in Ce3D™ Antibody Diluent Buffer. Each sphere was then treated with 500 μL of the diluted antibody solution and gently shaken at room temperature for 2 days. Following the primary antibody incubation, the spheres were washed three times with washing buffer, with each wash lasting 8 hours. Finally, the spheres were transferred to a confocal culture dish, and 500 μL of Ce3D™ clearing buffer was added to each sphere. The spheres were then incubated at room temperature for 4-6 hours until they became transparent. The stained and cleared spheres were observed using a Zeiss laser confocal microscope (LSM800). The dilution ratios for the primary antibodies were: PAX3 (Sigma, 1:100) and TYRP1 (Millipore, 1:200). The dilution ratio for all the secondary antibodies was 1:500.

#### Determination of melanin content

A total of 2×10^5^ cells were counted and centrifuged at 4,000g for 5 minutes. The cell pellet was washed twice with PBS to remove any residual culture medium. The cells were then dissolved in 250μL of 1M NaOH containing 10% dimethyl sulfoxide. The cell lysate was incubated at 80°C for 2 hours to solubilize the melanin. After the incubation, 100 μL of the cell lysate was transferred into a 96-well plate for absorbance measurement. The absorbance of the cell lysate was measured at a wavelength of 500 nm using a full-wavelength spectrophotometer (Thermo Multiskan SkyHigh). A standard curve was generated using synthetic melanin (Sigma) as the reference. The concentration of melanin in the samples was determined by reading the OD500nm against the standard curve.

#### Cell sorting

The cells were dissociated using TrypLE Express Enzyme and centrifuged at 1,000 rpm for 5 minutes. PDGFRB-PE (BD Biosciences) antibody was added to the dissociated cells. The recommended antibody concentration is 20 μL per 2×10^6^ cells. The cells were incubated with the antibody on ice in the dark for 30 minutes. After the incubation, the cells were washed with a buffer solution containing 1% FBS in PBS and they were then sorted using a BD FACS Aria flow cytometer.

#### Comparison of cell proliferation ability

The cells were seeded uniformly at a density of 6,000 cells per well in a 96-well plate, with three replicates for each experimental group. After 24 hours of cell attachment, 10 μL of the CCK-8 Cell Counting Kit (Vacyme) reagent was added to each well. The cells were incubated in a light-protected cell culture incubator at 37°C for 2 hours. After the incubation, the absorbance at 450 nm was measured using a full-wavelength spectrophotometer (Thermo Multiskan SkyHigh).

#### _L_-DOPA staining

The cells were fixed with 4% paraformaldehyde and then incubated in a 0.1% solution of l-DOPA (L-3,4-dihydroxyphenylalanine) prepared in PBS for 3-4 hours at 37°C. The nuclei were then counterstained with haematoxylin. Finally, the stained sections were thoroughly dehydrated in absolute alcohol.

#### Masson-Fontana staining

The cells were fixed with 4% paraformaldehyde and then treated with ammoniacal silver nitrate solution (from a Masson-Fontana staining kit) in a closed jar for 40 minutes at 56°C. The samples were thoroughly washed in distilled deionized water (ddH_2_O) and then treated with a hypo solution for 5 minutes. The samples were counterstained with neutral red stain for 5 minutes and rinsed in ddH_2_O. Finally, the stained preparations were thoroughly dehydrated. The stained images were analyzed using ImageJ to quantify melanin granule levels and calculate the melanin granule index. Cells were categorized based on staining intensity as follows: - (score 0), uniform light staining without visible granules; + (score 1), light brown granules; ++ (score 2), dark brown granules; +++ (score 3), brown-black granules. The melanin granule index is calculated by summing the category score of each cell in the image and then obtaining the average.

#### Single cell preparation for sequencing

The cells were dissociated into single cells using TrypLE digestion. After digestion, the cell suspension was filtered through a 40-micron cell strainer to remove any cell clumps. The cell suspension was centrifuged, and the supernatant was discarded. The cells were resuspended in cryopreservation solution (Stem Cell Baker). Cell counting was performed, and the cell concentration was adjusted to 5-10 million cells per millilitre. The cells suspension was aliquoted into cryovials and stored at -80°C overnight. After overnight storage, the cryovials were transferred to liquid nitrogen for long-term preservation.

#### Library preparation, sequencing and processing

The D23, D32, and D44 single-cell samples were prepared for sequencing as follows: Sample D23 was derived from an independent induction experiment and sequenced separately. Samples D32 and D44 were derived from the same induction experiment, harvested on different days (Day 32 and Day 44, respectively), and sequenced together in a single batch. The single-cell suspensions were shipped to BGI Genomics for library preparation using the DNBSEQ protocol. Sequencing was performed on the 10×Genomics platform. The FASTQ files generated from the single-cell RNA sequencing datasets were processed using Cell Ranger (v6.1.2, 10× Genomics), which aligned the reads to the GRCh38 reference genome and quantified gene expression. The datasets from the three time points were subsequently merged for downstream analysis. Further analysis was conducted in R (v4.2.2) and RStudio (v2022.07.1) using the Seurat package (v4.3.0).^33^

#### Filtering

To filter out low-quality cells and debris, cells with less than 3 detected genes and cells with more than 10% of UMIs (Unique Molecular Identifiers) mapped to mitochondrial gene were excluded. To exclude potential cell doublets and aggregates, retain cells with “nFeature_RNA” values between 1000 and 7000 for the D23 dataset, and between 3000 and 9000 for the D32 and D44 datasets. Additionally, retain cells with “nCount_RNA” values between 500 and 50,000 for the D23 dataset, and between 500 and 100,000 for the D32 and D44 datasets. After applying the filtering criteria, the common genes present in all the filtered datasets were used for further analysis.

#### Normalization

The UMI counts for each cell were log-normalized using the NormalizeData function in Seurat. The scale factor used for normalization was 10,000. FindVariableFeatures function was used to identify the most highly variable genes in the dataset with the “vst” (variance-stabilizing transformation) method.

#### Clustering

1. The expression of the highly variable genes identified was scaled.
2. Cell cycle status was predicted using the Seurat-provided gene sets for S phase and G2M Phase. The S.Score and G2M.Score variables were then regressed out using SCTransform function.
3. Principal component analysis (PCA) was performed using RunPCA function, with the “sct” assay and the first 50 principal components.
4. Batch correction was performed using the RunHarmony function from harmony package (version 1.1.0).^75^ The “sct” method and PCA as the reduction input were used, and the time points and batches were provided as the grouping variable. Shared nearest neighbor (sNN) graphs were constructed using the FindNeighbors function. Louvain clustering (resolution = 0.4) was then used to identify cell clusters via the FindClusters function and the cell clusters were visualized using two-dimensional Uniform manifold approximation and projection (UMAP) with the harmony correction.
5. The identities of the cell cluster were determined by assessing the expression and distribution of known marker genes.

#### Differential expression analysis

The RNA assay was chosen for subsequent analysis. Differentially expressed genes (DEGs) within the clusters were identified using the FindAllMarkers function, applying a log2-fold change threshold of 0.25 and a minimum expressing percentage of 0.25. The Wilcoxon Rank Sum test was used as the statistical method, and only genes with a p-value adjusted for multiple testing of less than 0.05 were selected as differentially expressed.

#### Gene enrichment analysis

The gene symbols were mapped to their corresponding Entrez Gene IDs, and enrichment analysis of Gene Ontology (GO) terms for biological processes (BP) was performed using the clusterProfiler (v4.6.0) R package.^76^ To identify pathway-derived subgroups, the analysis focused on developmental and signalling-related pathways from the KEGG database. Gene set enrichment analysis (GSEA) was then utilized with clusterProfiler and the msigdbr (v7.5.1) R package^77^ to further characterize the identified cell clusters (Figure 2G).

#### Trajectory analysis

Monocle (v2.26.0)^35^^,^^36^^,^^37^ was used to calculate the monocle trajectory for the cells that remained, via the detectGenes function (min_expr = 1). Variable genes, expressed in at least 10 cells, were analyzed using the SetOrderingFilter function for subsequent analysis. The distribution of the pseudo-time was calculated by the orderCells function and visualized by the plot_cell_trajectory function (Figure 3A). Furthermore, the differentialGeneSet function and BEAM function were used to identify genes associated with trajectory differentiation through differential gene expression analysis based on pseudo-time in single-cell trajectory analysis. Finally, the plot_genes_branched_heatmap function was employed to visualize the gene expression patterns in the context of the branched trajectory analysis, utilizing genes with a false discovery rate (q-value) < 1e-4 as input (Figure 3F; Table S3).

#### Comparison with human melanocytes dataset

The induced melanocyte datasets in this study were integrated with a human skin melanocyte dataset^41^ (GSE151091) using the integration method described by Stuart et al.^33^ with 15 anchors and first 50 principal components. The cell annotation was defined according to the literature, and the gene expression distributions were visualized using UMAP (Figures 4A and S3A). Differential expression analysis was conducted using the same method as before. Spearman correlation analysis was performed to compare cell types between the human melanocyte and induced melanocyte datasets, using the top 100 DEGs of each cluster, based on the average log2-fold change in the latter dataset. The average gene expression levels were obtained using the AverageExpression function with the “RNA” assay and “data” slot, and the ScaleData function was applied. The results were visualized using the pheatmap (v1.0.12) R package (Figures 4B and S3B; Table S6). Similarly, specific genes from the four developmental stages in the human melanocyte dataset were visualized on heatmap plots (Figures 4C, S3D and S4B; Tables S6 and S7), and gene module scores were calculated. Differential expression analysis and Venn diagram visualization were used to find common surface marker genes between the induced melanocyte (iMel) and human melanocyte (hMel) datasets (Figure S5A; Table S9). Based on the expression of surface marker genes, cells were divided into positive and negative clusters, and the two clusters were compared to identify upregulated and downregulated DEGs for GO-BP and KEGG pathway analysis (Figures 6B and S5C; Table S9). Additionally, the positive and negative proportions were counted, and bar plots were drawn, combined with other cell information (Figures 6C and S5B; Table S9).

#### Comparison between human and mouse melanocytes

A cross-species comparison was conducted using genes with a 1:1 ortholog between human and mouse, identified through the BiomaRt (v2.54.0)^78^ R package. The mouse gene names were subsequently converted to their human counterparts for consistency in the analysis. Mouse melanocyte datasets were incorporated from two studies by Sun et al.^16^^,^^45^ (GSE113502 and GSE203051). These datasets were integrated using a similar approach, utilizing common genes across the three source datasets (human, induced, and mouse melanocytes) for subsequent analysis. Pearson correlation analysis of cell types between the human melanocyte,^41^ induced melanocyte, and mouse melanocyte datasets was performed using these common genes across the three datasets (Figure S4C). Additionally, Spearman correlation analysis of subclusters within the three melanocyte sources was conducted using the top 100 differentially expressed genes of each cluster, based on the average log2-fold change in the human melanocyte dataset (Figure S3C). The cell groupings were classified as follows: in the induced melanocyte (iMel) datasets, iMel_C2 and iMel_C3 were categorized as undifferentiated (unDiff), while iMel_C1 was classified as differentiated (Diff). In the mouse melanocyte (mMel) datasets, mMel_TeSC and mMel_EaSC were grouped as undifferentiated (unDiff), and mMel_Diff was categorized as differentiated (Diff). For the human melanocyte (hMel) dataset, hMel_ADT, hMel_NEO, and hMel_FET were classified as differentiated, whereas hMel_STEM was categorized as undifferentiated. Heatmap analysis was performed using the melanocyte-related genes from GO-BP terms containing “pigment” or “melanin” between the undifferentiated and differentiated melanocytes across the three source datasets (Figure S4D; Table S7). Finally, Venn diagrams were used to illustrate the overlap of significantly upregulated DEGs of undifferentiated or differentiated melanocytes across the three datasets, respectively (Figure S4E; Table S7).

#### Gene module scores

Custom gene scores were calculated using the AddModuleScore function in Seurat (v4.3.0) to assess the expression of genes associated with melanocyte cluster identity (Figures 2E and S4A) and developmental stage signatures (Figures 4E, S3E and S4A). These scores provided insights into the molecular characteristics and differentiation potential of the identified clusters.

Differentiation scores were derived from the gene set reported by Bajpai et al.,^34^ which identified 169 genes involved in melanosome biogenesis, endosomal transport, and gene regulation, highlighting their essential roles in melanogenesis. Of these, 151 genes overlapped with the expressed genes in both the iMel and hMel datasets and were used to compute differentiation score (Figure 2E; Table S2). Similarly, 147 genes were identified in the mMel dataset as the intersection of genes expressed in all three datasets (iMel, hMel, and mMel) (Figure S4A; Table S7). These genes were utilized to assess melanocyte differentiation status within the system.

Stemness scores were calculated based on the 100 specific genes enriched in human melanocyte stem cells program identified by Belote et al.^41^ Of these, 90 genes overlapped with the expressed genes in both the iMel and hMel datasets, and were used to compute stemness score for iMel cell clusters. For the mMel dataset, 80 genes were identified as the intersection of the stemness genes set with the expressed genes in all three datasets (iMel, hMel, and mMel) (Figure S4A; Table S7). Additionally, genes from the enriched GO-BP terms of interest were scored to provide further insights into the functional characteristics of the cell populations (Figure S1G; Table S2).

#### Probabilistic cell-cell signalling networks

Cell-cell communication analysis for the induced melanocyte datasets was performed using the R package CellChat (v1.6.1).^49^ Interaction quantity, representing the frequency of signalling events, and interaction strength were quantified between cell clusters. Interaction strength reflects the cumulative effect of signalling pathways and was calculated by integrating the contribution of each ligand-receptor pair within a pathway (Figure 5B). This metric provides a weighted measure of the overall impact of intercellular communication. The arrows in the plot indicate the direction of signal transmission, and the thickness of the lines represents either the interaction quantity or strength. Key pathways were identified through DEGs analysis, mapped to KEGG pathways, and visualized using dot plots (Figure 5A; Table S8). Directional signalling networks were inferred, with arrows indicating the flow of communication and line thickness scaled by interaction quantity or strength (Figure 5C). Network centrality measures were computed for ncWNT, KIT, TGF-β, and BMP signalling, highlighting the relative importance of each cluster (Figure 5D). Ligand-receptor interactions contributing to these pathways were further analyzed and visualized through dot and violin plots, showing expression levels and communication probabilities (Figures 5E and 5F).

### Quantification and statistical analysis

Statistical and graphical data analyses were performed using Microsoft Excel, GraphPad Prism 8, and R (version 4.2.2) software. Data are presented as means ± standard deviation (SD). Statistical tests were applied as follows: melanin content data were analyzed using Student’s t-test, while melanin granule index data from Masson-Fontana staining were analyzed using one-way ANOVA with multiple comparisons. For violin plots in scRNA-seq analyses, p-values were calculated using one-sided Wilcoxon rank-sum tests and are displayed on the plots. (∗p < 0.05, ∗∗p < 0.01, ∗∗∗p < 0.001, ∗∗∗∗p < 0.0001). Details are described in the figure legends. Data are representative of three independent experiments.

## Supplemental information

Document S1. Figures S1–S5 and Table S1

Table S2. Differential expression and enrichment analysis of the three iMel clusters, related to Figures 2 and S1 (A) Upregulated DEGs of the three clusters, related to Figure 2D. (B) Genes involved in the calculation of differentiation score, related to Figure 2E. (C) GO-BP terms from the upregulated DEGs of the three clusters, related to Figure 2F. (D) Average expression of NCC and melanocyte marker genes for the three clusters, related to Figure 2H. (E) Gene involved in the calculation of neural-related GO-BP term scores, related to Figure S1G.

Table S3. Functional enrichment and marker gene expression across differentiation time points and pseudo-time trajectory, related to Figure 3 (A) GO-BP terms enriched from upregulated DEGs of the three time point groups, related to Figure 3C. (B) Average expression of NCC and melanocyte marker genes for the three time point groups, related to Figure 3D. (C) GO-BP terms related to pigmentation from pseudo-time analysis, related to Figure 3F.

Table S4. Analysis of iMel clusters based on early embryonic neural markers, related to Figures 3 and S2 (A) Average expression of human early embryonic neural genes across the three clusters, related to Figure 3G. (B) GO-BP terms enriched in upregulated DEGs from NTCs compared to pre-EMT NCCs, related to Figure S2C. (C) Average expression of genes enriched in the “axon development” (GO:0061564) GO-BP term for the four identified cell types, related to Figure S2D. (D) Average expression of genes enriched in the “neural precursor cell proliferation” (GO:0061351) GO-BP term for the four identified cell types, related to Figure S2E. (E) Average expression of genes enriched in the “regulation of neurogenesis” (GO:0050767) GO-BP term for the four identified cell types, related to Figure S2F. (F) Average expression of genes enriched in the “forebrain development” (GO:0030900) GO-BP term for the four identified cell types, related to Figure S2G.

Table S5. Cell identity in iMel, related to Figures 3 and S2 (A) Proportions of cells in C1, C2, and C3 based on co-expression of collagen and melanocyte-related genes, related to Figure 3I. (B) Proportions of cells in C1, C2, and C3 based on co-expression of collagen and melanocyte-related genes, related to Figure S2J. (C) Expression of human skin cell markers in iMel and hMel, related to Figure S2K.

Table S6. Gene expression and correlation analysis of iMel and hMel subclusters, related to Figures 4 and S3 (A) The top 100 genes from each cluster of hMel used in the Spearman correlation analysis, related to Figure 4B and Figure S3B. (B) Expression of specific genes from four hMel developmental stages in iMel clusters, related to Figure 4C. (C) Expression of melanocyte stem cell markers in three iMel clusters, related to Figure 4D. (D) Genes involved in the calculation of stemness scores based on melanocyte stem cell from hMel datasets, related to Figure 4E. (E) Expression of the conserved genes across iMel and hMel, related to Figure 4F. (F) Expression of hMel developmental stage-specific genes in the C1 subclusters of iMel, related to Figure S3D. (G) Upregulated genes involved in score calculation across the four stages of hMel in the C1 subclusters of iMel, related to Figure S3E. (H) Upregulated DEGs from the C1 subclusters involved in the top GO-BP analysis, related to Figure S3F. (I) GO-BP terms using the conserved genes from Figure 4F, related to Figure S3G.

Table S7. Comparative analysis across iMel, hMel, and mMel, related to Figure S4 (A) Genes involved in calculating the melanocyte differentiation (left) and stemness (right) scores in mMel, related to Figure S4A. (B) Expression of hMel developmental stage-related genes in mMel, related to Figure S4B. (C) Expression of melanocyte-related genes in undifferentiated and differentiated melanocytes across iMel, mMel, and hMel, related to Figure S4D. (D) Genes involved in the Venn diagrams of undifferentiated and differentiated melanocytes across iMel, mMel, and hMel, related to Figure S4E.

Table S8. Common KEGG pathways enriched by DEGs from iMel_C2 and hMel_STEM, related to Figure 5A

Table S9. Surface marker-related analyses in iMel and hMel, related to Figures 6 and S5 (A) Expression of enriched surface markers in iMel and hMel, related to Figure 6A. (B) GO-BP terms for upregulated DEGs in surface marker-positive hMel clusters, related to Figure 6B. (C) GO-BP terms for downregulated DEGs in surface marker-positive hMel clusters, related to Figure 6B. (D) Fraction of cells expressing positive or negative surface marker genes in iMel, related to Figure 6C. (E) Upregulated genes in iMel_C2 and hMel_STEM involved in the Venn diagram, related to Figure S5A. (F) Fraction of cells expressing positive or negative surface marker genes in hMel (left) and iMel (right) datasets, related to Figure S5B. (G) KEGG terms for upregulated DEGs in surface marker-positive clusters of hMel, related to Figure S5C (left). (H) KEGG terms for downregulated DEGs in surface marker-positive clusters of hMel, related to Figure S5C (right).
