# Supplementary material for: Insights into human melanocyte development and characteristics through pluripotent stem cells combined with single-cell sequencing
Source: iScience. 2025 Apr 8;28(5):112373. doi: 10.1016/j.isci.2025.112373 (PMC12245449; doi:10.1016/j.isci.2025.112373)
Supplement: Document S1. Figures S1–S5 and Table S1 [file mmc1.pdf]

iScience, Volume *28*

Supplemental information

Insights into human melanocyte development and characteristics through pluripotent stem cells combined with single-cell sequencing

Jie Yang, Zihan Wang, Hang Zhou, Yuyun Xiong, Yumei Li, Yun-wen Zheng, and Liping Liu

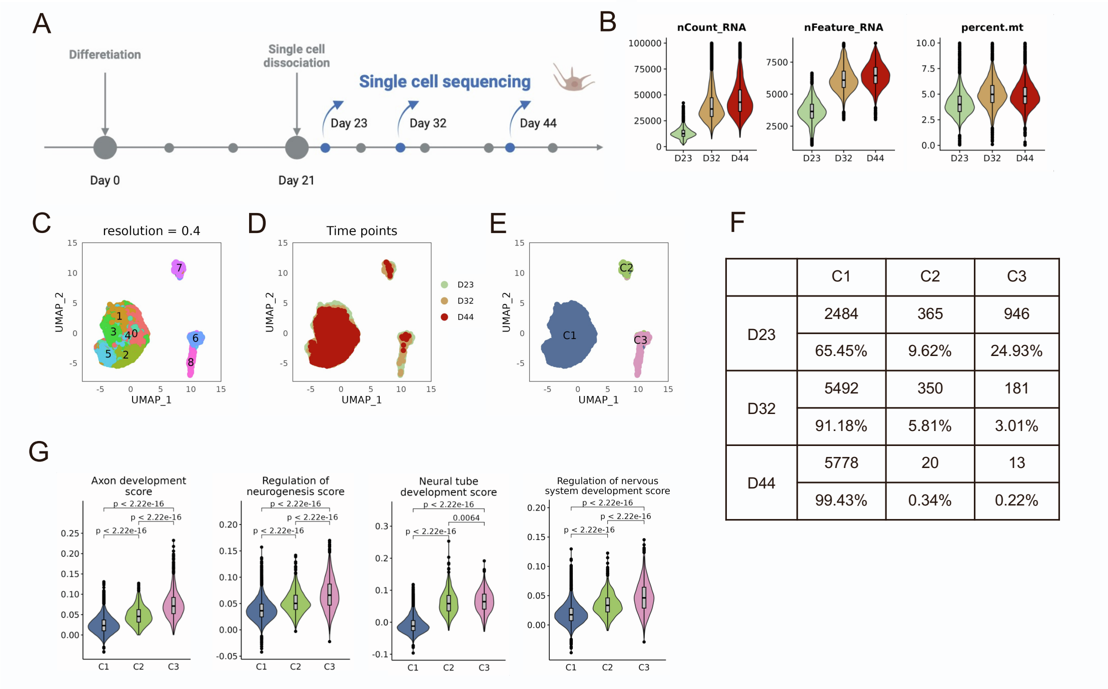

	C1	C2	C3
D23	2484	365	946
	65.45%	9.62%	24.93%
D32	5492	350	181
	91.18%	5.81%	3.01%
D44	5778	20	13
	99.43%	0.34%	0.22%

Figure S1. scRNA-seq and analysis of induced melanocytes. Related to Figure 2.

(A) Schematic of sampling of single-cell sequencing.

(B) Quality control plots showing the number of counts, number of genes, and percentage of mitochondrial genes found for the cells in each of the three induced melanocyte datasets.

(C) UMAP plot of unsupervised clustering. Each dot represents a cell colored by its assigned cluster.

(D) UMAP plot of the three time points. Each dot represents a cell colored by the time points.

(E) UMAP plot of the three cell clusters. Each dot represents a cell colored by the cell clusters.

(F) Table displaying the numbers and proportions of each cell cluster in each dataset at three time points.

(G) Violin plots indicating the scores of neural related biological process terms in Gene Ontology in the three clusters. p-values are displayed on the plot using one-sided Wilcoxon rank-sum tests.

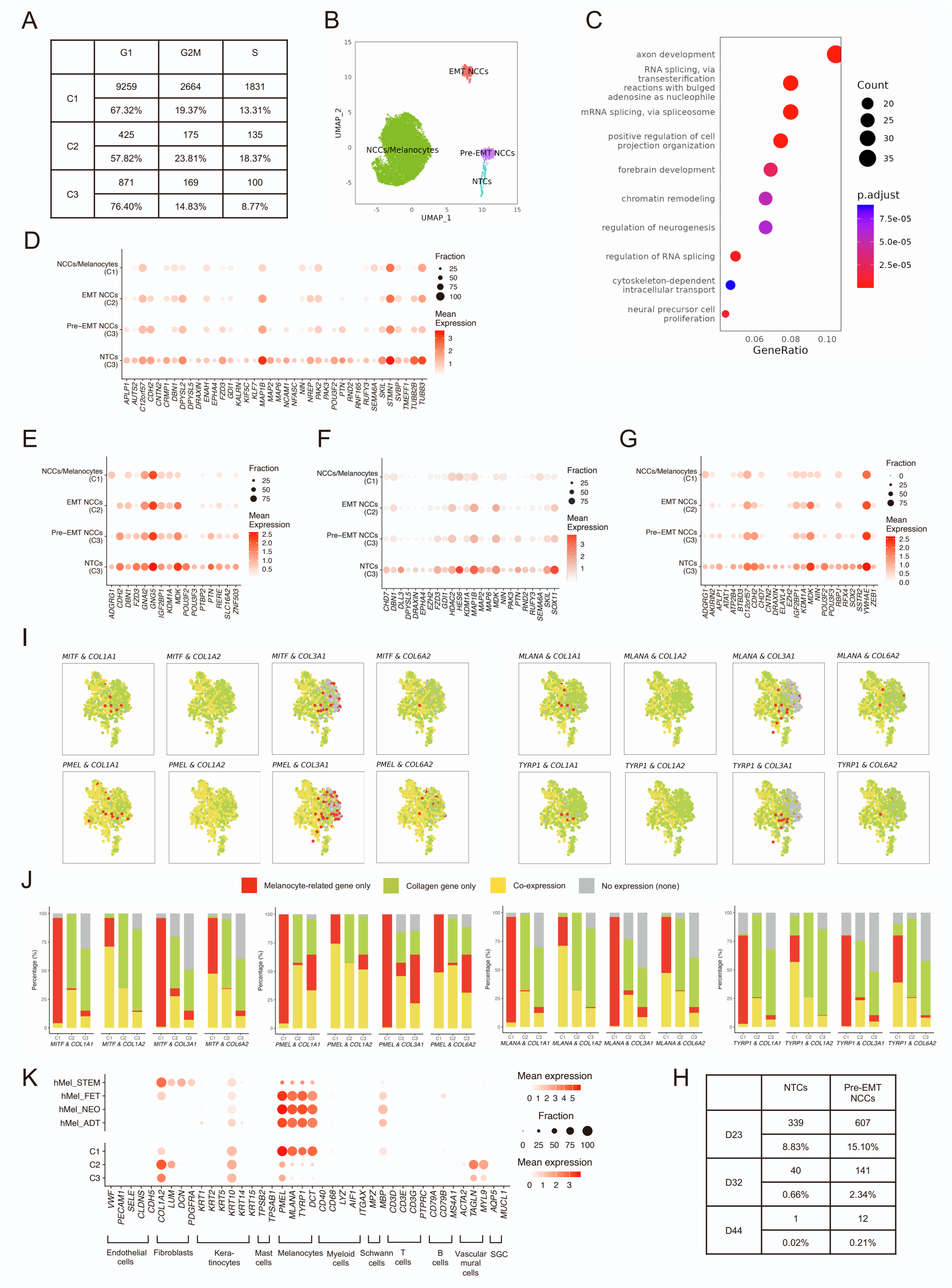

A

		G1	G2M	S
C1		9259	2664	1831
		67.32%	19.37%	13.31%
C2		425	175	135
		57.82%	23.81%	18.37%
C3		871	169	100
		76.40%	14.83%	8.77%

H

	NTCs	Pre-EMT NCCs
D23	339	607
	8.83%	15.10%
D32	40	141
	0.66%	2.34%
D44	1	12
	0.02%	0.21%

Figure S2. Supplementary Analysis of the Dynamic Differentiation Trajectory of Induced Melanocytes. Related to Figure 3.

(A) Table displaying the numbers and proportions of each cell cycle phase in each cell cluster.

(B) UMAP plot of induced melanocytes colored by cell identity of Figure 3G. NTCs, neural tube cells; Pre-EMT NCCs, pre-epithelial-to-mesenchymal transition neural crest cells; EMT NCCs, epithelial-to-mesenchymal transition neural crest cells; NCCs, neural crest cells.

(C) Dot plot showing the top 10 biological process (BP) terms in Gene Ontology (GO) for genes upregulated in NTCs, comparing to Pre-EMT NCCs. The size and color of each dot represent the gene count and significance level, respectively.
(D) Dot plot showing the expression of marker genes from axon development GO-BP term in the four cell clusters.
(E) Dot plot showing the expression of marker genes from neural precursor cell proliferation GO-BP term in the four cell clusters.
(F) Dot plot showing the expression of marker genes from regulation of neurogenesis GO-BP term in the four cell clusters.
(G) Dot plot showing the expression of marker genes from forebrain development GO-BP term in the four cell clusters.
(H) Table displaying the numbers and proportions of NTCs and Pre-EMT NCCs of C3 at three time points.
(I) Enlarged UMAP plots of the C2 cluster. Cells are colored as follows: red indicates expression of the melanocyte-related gene, green indicates expression of the collagen gene, yellow indicates co-expression of both, and gray indicates no expression.
(J) Bar plots summarizing the proportions of cells in C1, C2, and C3: gray (none), green (collagen only), red (melanocyte only), and yellow (co-expression).
(K) Dot plot showing the relative expression of human skin cell markers [S1] in induced melanocyte (iMel) and human melanocyte (hMel) dataset [S2].

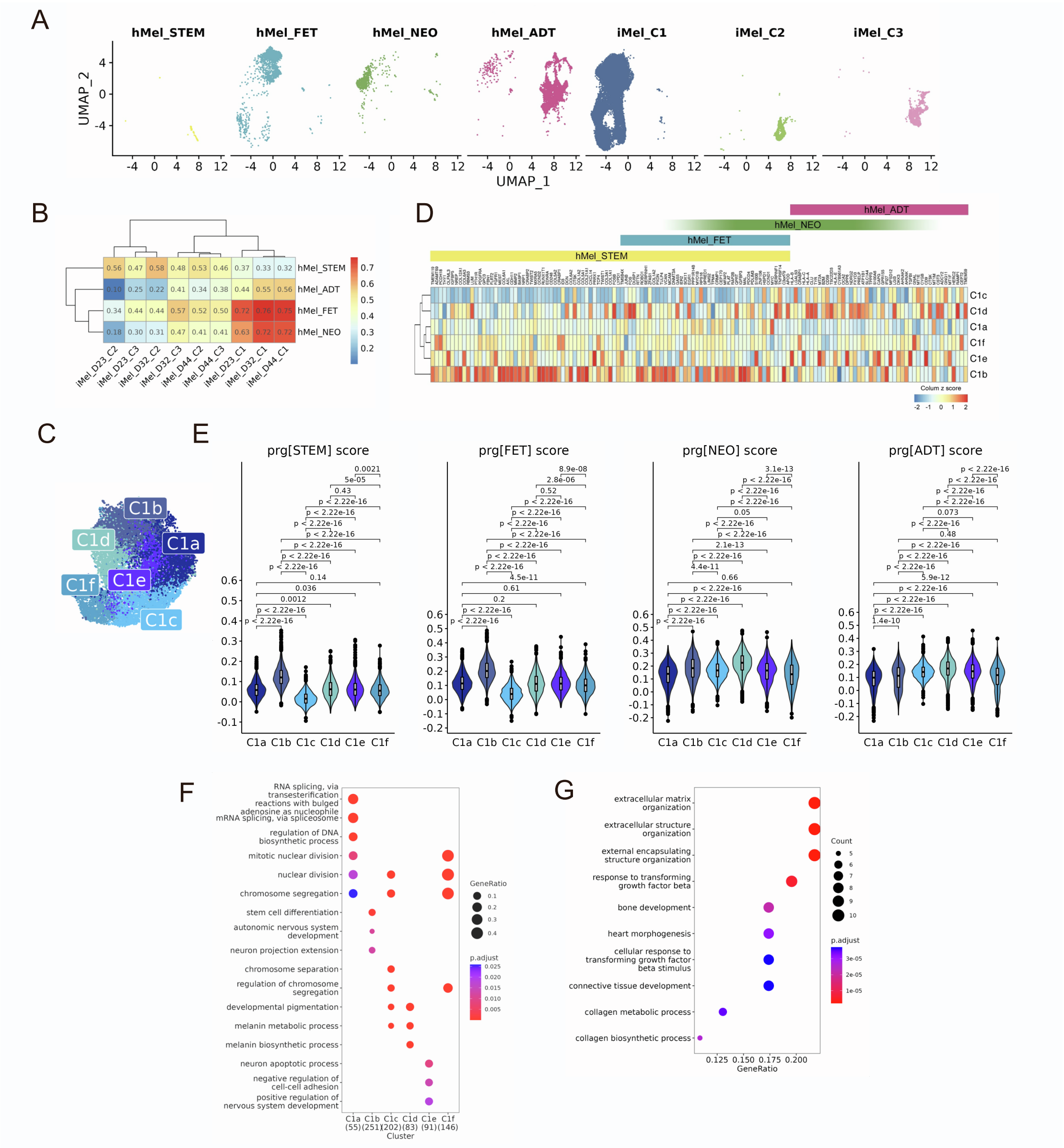

Figure S3. Similarity between induced melanocytes and human melanocytes. Related to Figure 4.

(A) UMAP plot of Figure 4A split by the cell identities.

(B) Spearman correlation analysis for each cluster within cell types and time points of differentiation between induced melanocyte (iMel) and human melanocyte (hMel) using the same genes as Figure 4B.

(C) UMAP plot of C1 subclusters split by the cell identities (Figure S1C).

(D) Heat map of the relative expression (column z score) for the C1 subclusters of iMel using the same genes as Figure 4C.

(E) Violin plots indicating the scores calculated by the upregulated genes of four stages of human melanocytes for the C1 subclusters in iMel.

(F) Dot plot showing the top three biological process terms for upregulated genes in each C1 subcluster. The numbers of selected genes are shown at the bottom. The size and color of each dot represent the gene ratio and significance level, respectively.

(G) Dot plot of top 10 GO-BP terms for the conserved genes shown in Figure 4F.

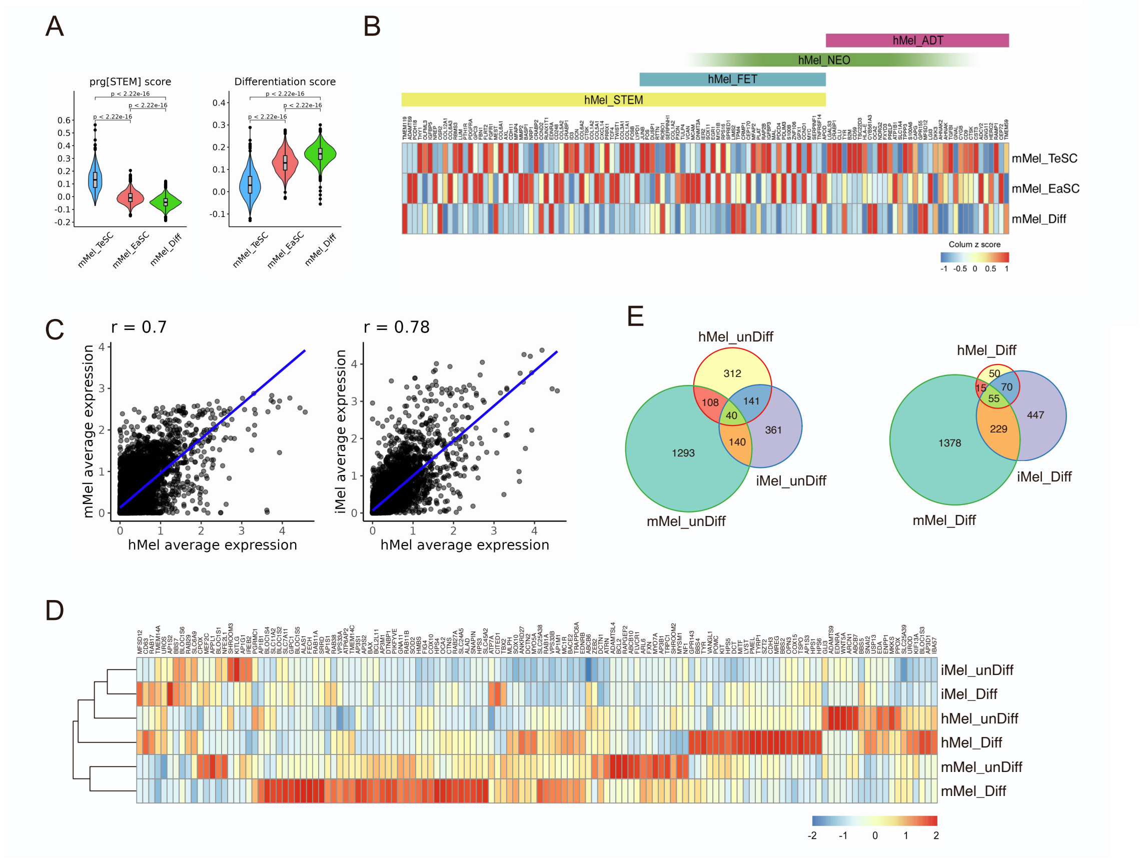

Figure S4. Comparative analysis of human, mouse, and induced melanocytes. Related to Figure 4.

(A) Violin plot showing the stemness score [S2] (left) and differentiation score [S3] (right) of three clusters of mouse melanocytes (mMel). TeSC, telogen melanocyte stem cells; EaSC, early-anagen melanocyte stem cells; Diff, differentiated melanocytes.

(B) Heat map indicating the relative expression (column z score) of specific genes identified from four developmental stages of human melanocyte (hMel) in mMel. STEM, melanocyte stem cells; FET, fetal melanocytes; NEO, neonatal melanocytes; ADT, adult melanocytes.

(C) Pearson correlation analysis between hMel and mouse melanocyte (mMel) (left) as well as between hMel and induced melanocyte (iMel) (right) using common genes within three sources. Each point represents a gene. The blue regression line indicates the linear relationship between the average gene expression levels of hMel and iMel (or mMel).

(D) Heat map of the relative expression (row z score) of melanocyte-related genes in undifferentiated (unDiff) and differentiated (Diff) melanocytes with three sources. Hierarchical clustering is applied on the left.

(E) Venn diagrams illustrating the overlap of upregulated DEGs in undifferentiated (unDiff, left) and differentiated (Diff, right) melanocytes across the three datasets, respectively.

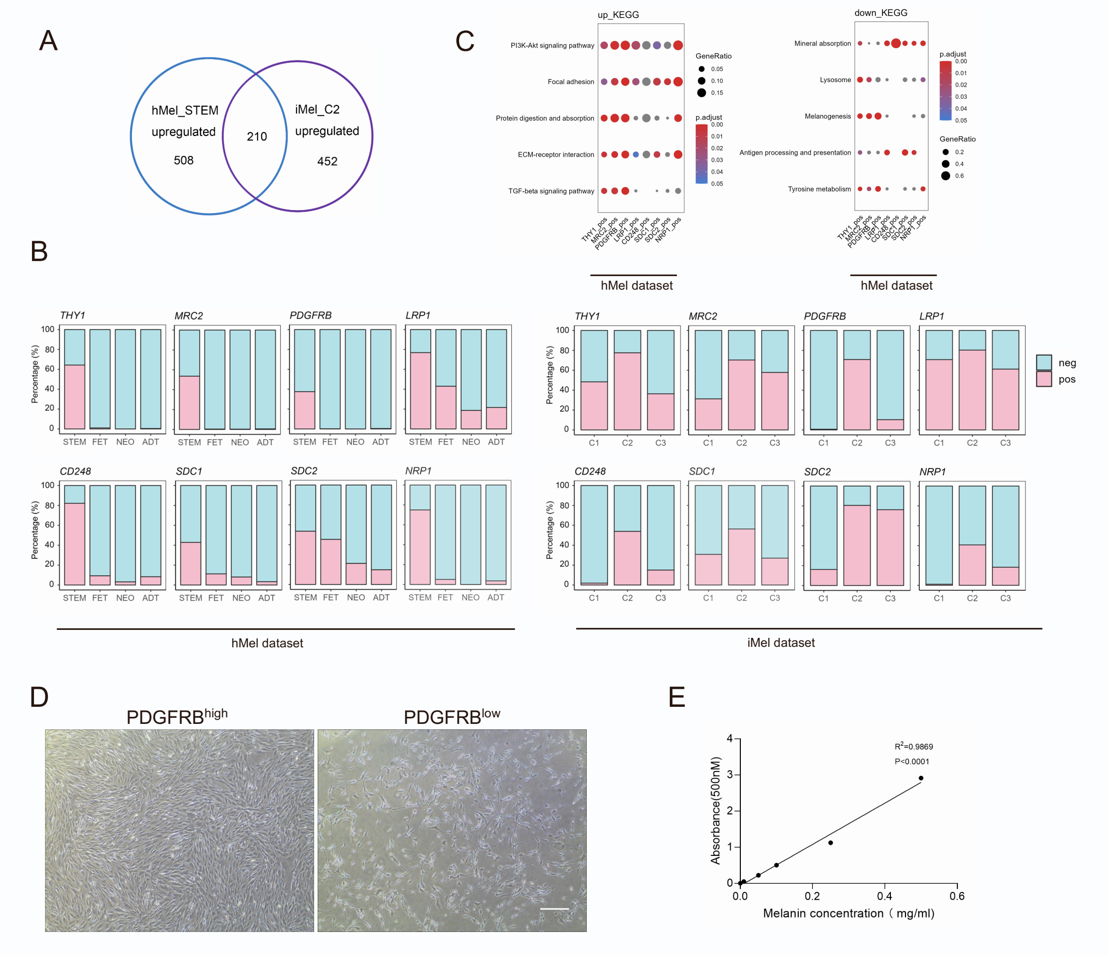

Figure S5. Surface marker screening for melanocyte stem cells. Related to Figure 6.

(A) Venn diagram illustrating the overlap of significantly upregulated DEGs in stem cells from human melanocyte (hMel) datasets and the C2 cluster from induced melanocyte (iMel) datasets.

(B) Bar plots showing the proportion of surface markers based on the expression for cell types in the hMel and iMel datasets, respectively. neg, negative; pos, positive.

(C) Dot plots showing the selected specific KEGG pathways for the upregulated and downregulated DEGs of the positive clusters in hMel, based on the gene expression of surface markers. pos, positive.

(D) Culture of induced melanocytes for 8 days after sorting. scale bar, 100 μm.

(E) Standard curve for melanin content determination.

Table S1. Real-time PCR primers used in this study. Related to STAR Methods.

REAGENT or RESOURCE	SOURCE	IDENTIFIER
Oligonucleotides		
GAPDH<br>F: 5′-ATGTTCGTCATGGGTGTGAA-3′<br>R: 5′-TGTGGTCATGAGTCCTTCCA-3′	Sangon Biotech, Shanghai	N/A
CDH2<br>F: 5′-CGCCAGGCCAAACAACTTTTAAT-3′<br>R: 5′-TGGCTCAAGTCATAGTCCTGGTC-3′	Sangon Biotech, Shanghai	N/A
MAP2<br>MAP2 F:5'-CAGTTTCTGCGCCCAGATTT-3'<br>MAP2 R:5'-CCCAATCAATGCTTCCTCGG-3'	Sangon Biotech, Shanghai	N/A
NCAM1<br>F: 5′-GTTCATGTGCATTGCGGTCA-3′<br>R: 5′-TTGGACTCATCTTTCGAGAAGGC-3′	Sangon Biotech, Shanghai	N/A
PAX6<br>F: 5′-CCATCACCAATCAGCATAGGAAT-3′<br>R: 5′-AAAGGAGTTGCTGGTGAGAGT-3′	Sangon Biotech, Shanghai	N/A
TUBB3<br>F:5′-TCTTGAGAGAAAAGAGAGAGACAGG-3′<br>R: 5′-TCACTGATGACTTCCCAGAACTGT-3′	Sangon Biotech, Shanghai	N/A
NES<br>F: 5′-TCCCTCAGCTTTCAGGACCC-3′<br>R: 5′-GCTCAGGACTGGGAGCAAAG-3′	Sangon Biotech, Shanghai	N/A
PAX3<br>F: 5′-AGCCGCATCCTGAGAAGTAA-3′<br>R: 5′-CTTCATCTGATTGGGGTGCT-3′	Sangon Biotech, Shanghai	N/A
SOX10<br>F: 5′-GCTGGACAGAGGAGAAGGAGGT-3′<br>R: 5′-GAAAACAGGAGTCATAGGAGTGGTA-3′	Sangon Biotech, Shanghai	N/A
MITF<br>F: 5′-AGACATGCGCTGGAACAAG-3′<br>R: 5′-ACCCGTGGATGGAATAAGG-3	Sangon Biotech, Shanghai	N/A
PMEL<br>F: 5′-GGGCTACAAAAGGGAGCCAG-3′<br>R: 5′-AAGGTCCACCATCAGGGAAGA-3′	Sangon Biotech, Shanghai	N/A
MLANA<br>F: 5′-GCACTCAATGTGCCTTAACAAGAA-3′<br>R: 5′-TGGAGCATTGGGAACCACAGG-3′	Sangon Biotech, Shanghai	N/A
TYR<br>F: 5′-TGCACAGAGAGACGACTCTTG-3′<br>R: 5′-GAGCTGATGGTATGCTTTGCTAA-3′	Sangon Biotech, Shanghai	N/A

TYRP1<br>F: 5′-CCCTGGATATGGCAAAGCG-3′<br>R: 5′-CCTGTCCTACCCCAAGGAAAG-3′	Sangon Biotech, Shanghai	N/A
DCT<br>F: 5′-AACTGCGAGCGGAAGAAACC-3′<br>R: 5′-CGTAGTCGGGGTGTACTCTCT-3′	Sangon Biotech, Shanghai	N/A
THY1<br>F:5'-TGGGGATGGCGAGTGACTTA-3'<br>R:5'-CCTGGATCGGGTTATGATGGG-3'	Sangon Biotech, Shanghai	N/A
MRC2<br>F: 5′-GTGTTTGTCTGGGAGCACCT-3′<br>R: 5′-GACCAGAGTGCCTCCTTTGG-3′	Sangon Biotech, Shanghai	N/A
PDGFRB<br>F: 5′-CCATCAGCAGCAAGGCGA-3′<br>R: 5′-CCCGGGGGTGTGACGA-3′	Sangon Biotech, Shanghai	N/A
EDNRA<br>F: 5′-GGTGTGCACTGCGATCTTCT-3′<br>R: 5′-CCACTTCTCGACGCTGCTTA-3′	Sangon Biotech, Shanghai	N/A
SMAD1<br>F: 5′-CATGACCCAGGATGGCTCTC-3′<br>R: 5′-CAACCGCCTGAACATCTCCT-3′	Sangon Biotech, Shanghai	N/A
TWIST2<br>F: 5′-GCTACAGCAAGAAGTCGAGCGAAGA-3′<br>R: 5′-TTGTCAGAGGGCAGCGTGGG-3′	Sangon Biotech, Shanghai	N/A
BMP4<br>F: 5′-GGGCTTCCACCGTATAAACA-3′<br>R: 5′-GGACCAGTCTCGTGTCCAGT-3′	Sangon Biotech, Shanghai	N/A
SNAI1<br>F: 5′-TCGGAAGCCTAACTACAGCGA-3′<br>R: 5′-AGATGAGCATTGGCAGCGAG-3′	Sangon Biotech, Shanghai	N/A

Supplemental references:

[S1] Chen, M., Yang, L., Zhou, P., Jin, S., Wu, Z., Tan, Z., Xiao, W., Xu, S., Zhu, Y., Wang, M., et al. (2024). Single-cell transcriptomics reveals aberrant skin-resident cell populations and identifies fibroblasts as a determinant in rosacea. Nat Commun *15*, 8737. https://doi.org/10.1038/s41467-024-52946-7.

[S2] Belote, R.L., Le, D., Maynard, A., Lang, U.E., Sinclair, A., Lohman, B.K., Planells-Palop, V., Baskin, L., Tward, A.D., Darmanis, S., et al. (2021). Human melanocyte development and melanoma dedifferentiation at single-cell resolution. Nat Cell Biol *23*, 1035–1047. https://doi.org/10.1038/s41556-021-00740-8.

[S3] Bajpai, V.K., Swigut, T., Mohammed, J., Naqvi, S., Arreola, M., Tycko, J., Kim, T.C., Pritchard, J.K., Bassik, M.C., and Wysocka, J. (2023). A genome-wide genetic screen uncovers determinants of human pigmentation. Science *381*, eade6289. https://doi.org/10.1126/science.ade6289.
